# Neoadjuvant immunotherapy and chemotherapy regimens for the treatment of high-risk, early-stage triple-negative breast cancer: a systematic review and network meta-analysis

**DOI:** 10.1186/s12885-023-11293-4

**Published:** 2023-08-23

**Authors:** Javier Cortes, Amin Haiderali, Min Huang, Wilbur Pan, Peter Schmid, Katherine G. Akers, Julie E. Park, Andrew M. Frederickson, Peter A. Fasching, Joyce O’Shaughnessy

**Affiliations:** 1 Oncology Department, International Breast Cancer Center, Pangaea Oncology, Quiron Group, Barcelona, Spain; 2 Medica Scientia Innovation Research, Barcelona, Spain; 3 Faculty of Biomedical and Health Sciences, Department of Medicine, Universidad Europea de Madrid, Madrid, Spain; 4 Merck & Co., Inc, Rahway, NJ USA; 5 Barts Cancer Institute, Queen Mary University of London, London, UK; 6 PRECISIONheor, New York, NY USA; 7 PRECISIONheor, Vancouver, BC Canada; 8 Department of Gynecology and Obstetrics, Comprehensive Cancer Center Erlangen, EMN, University Hospital Erlangen, Friedrich-Alexander University Erlangen, Nuremberg, Erlangen Germany; 9 Baylor University Medical Center, Texas Oncology and US Oncology, Dallas, TX USA

**Keywords:** Triple-negative breast cancer, Chemotherapy, Immunotherapy, Neoadjuvant therapy, PD-1, PD-L1, Network meta-analysis

## Abstract

**Background:**

Patients with triple-negative breast cancer (TNBC) are generally younger and more likely to experience disease recurrence and have the shortest survival among all breast cancer patients. Recently, neoadjuvant delivery of the programmed cell death protein-1 inhibitor pembrolizumab plus chemotherapy followed by adjuvant pembrolizumab was approved for patients with high-risk, early-stage TNBC, but this treatment regimen has not been evaluated in head-to-head trials with other neoadjuvant treatment regimens. Therefore, the objective of this study was to estimate the relative efficacy of neoadjuvant pembrolizumab + chemotherapy followed by adjuvant pembrolizumab versus other neoadjuvant treatments for early-stage TNBC through a systematic review and network meta-analysis (NMA).

**Methods:**

EMBASE, MEDLINE, Cochrane Central Register of Controlled Trials, conference abstracts, and clinical trial registries were searched for randomized controlled trials evaluating neoadjuvant treatments for early-stage TNBC. NMA was performed to estimate relative treatment effects among evaluated interventions.

**Results:**

Five trials met the inclusion criteria and were included in the NMA. The relative efficacy of neoadjuvant pembrolizumab + chemotherapy followed by adjuvant pembrolizumab was favorable to paclitaxel followed by anthracycline + cyclophosphamide in terms of pathologic complete response (pCR), event-free survival (EFS), and overall survival; paclitaxel + carboplatin followed by anthracycline + cyclophosphamide in terms of pCR and EFS; paclitaxel + bevacizumab followed by anthracycline + cyclophosphamide + bevacizumab in terms of pCR; and paclitaxel + carboplatin + veliparib followed by anthracycline + cyclophosphamide in terms of EFS.

**Conclusions:**

Neoadjuvant pembrolizumab + chemotherapy followed by adjuvant pembrolizumab confers benefits in response and survival outcomes versus alternative neoadjuvant treatments for early-stage TNBC.

**Supplementary Information:**

The online version contains supplementary material available at 10.1186/s12885-023-11293-4.

## Background

Breast cancer is the most frequently diagnosed cancer, with an estimated 2.3 million new cases worldwide in 2020, and the leading cause of cancer death among women [1]. Approximately 15–20% of breast cancer cases are molecularly classified as triple-negative breast cancer (TNBC), which is characterized by tumors lacking expression of the estrogen receptor, progesterone receptor, and epidermal growth factor receptor 2 [2, 3]. Compared with women with other molecular subtypes of breast cancer, women with TNBC generally present at younger ages and are more likely to experience early recurrence after treatment and distant metastasis to visceral organs including the brain [3–5]. Moreover, women with TNBC exhibit the shortest survival time among all breast cancer patients, with a mortality rate of 40% within 5 years of diagnosis [3, 5].

As TNBC is insensitive to endocrine therapy due to its molecular phenotype, cytotoxic chemotherapy has historically been the mainstay treatment approach. Chemotherapy combinations involving doxorubicin, cyclophosphamide, anthracycline, paclitaxel, or platinum-based agents administered before tumor resection are shown to improve early outcomes, including pathologic complete response (pCR) and event-free survival (EFS), among women with early-stage or locally advanced TNBC [3]. Advances in our understanding of the molecular mechanisms of cancer, however, have led to the development of new targeted therapies that may achieve even greater improvements in TNBC patient outcomes. Of particular interest, immunotherapies that inhibit immune checkpoints such as programmed cell death protein 1 (PD-1) or programmed cell death ligand 1 (PD-L1), which are leveraged by tumor cells to evade recognition and destruction by the immune system, can restore the body’s ability to effectively attack tumors. Indeed, neoadjuvant delivery of the PD-1 inhibitor pembrolizumab + chemotherapy followed by adjuvant pembrolizumab was granted U.S. Food and Drug Administration approval in July 2021 for treating high-risk, early-stage TNBC based on statistically significant and clinically meaningful improvements in pCR and EFS in a phase III randomized controlled trial (RCT) [6, 7]. Furthermore, another phase III RCT shows that neoadjuvant treatment with the PD-L1 inhibitor atezolizumab + chemotherapy improves pCR among early-stage TNBC patients [8].

Network meta-analysis (NMA) is a statistical method that enables indirect comparisons between treatments where head-to-head evidence may not be available, which allows clinicians, guideline developers, and health technology assessment agencies to evaluate evidence on new treatments within the context of all existing evidence [9, 10]. Specifically, NMA can be used to combine direct and indirect evidence regarding any interventions that form a connected network of RCTs wherein each trial has at least one intervention (active or placebo) in common with another trial and all trials are sufficiently similar [11]. Clinical trial evidence suggests that neoadjuvant pembrolizumab + chemotherapy followed by adjuvant pembrolizumab is an effective and safe approach to treating early-stage or locally advanced TNBC; however, this treatment regimen has not been compared against all alternative neoadjuvant treatment regimens in head-to-head trials. As the relative efficacy of various immunotherapy- and chemotherapy-based regimens is of interest to both clinicians and healthcare policymakers, the aim of this analysis was to estimate the comparative efficacy of neoadjuvant pembrolizumab + chemotherapy followed by adjuvant pembrolizumab versus other neoadjuvant treatments for patients with high-risk, early-stage TNBC in terms of pCR, EFS, and overall survival (OS) through a systematic review and NMA.

## Methods

### Systematic review

The systematic review and NMA were performed in accordance with Preferred Reporting Items for Systematic Reviews and Meta-analysis (PRISMA) guidelines [12] and followed a pre-specified protocol. Selection criteria for the population, interventions, comparators, outcomes, and study design (PICOS) are outlined in Table 1. RCTs were included if they enrolled patients with early-stage and locally recurrent non-metastatic TNBC, evaluated interventions of interest, reported outcomes of interest, and were published in English. Due to an anticipated lack of trials conducted solely in TNBC patients, trials were eligible for inclusion if they enrolled TNBC patients exclusively and reported at least one outcome of interest or if they enrolled patients from a broader population of breast cancer patients and reported at least one outcome of interest in a subgroup composed of > 90% TNBC patients. As the primary purpose of this systematic review and NMA was to identify and synthesize evidence from the clinical literature to support health technology assessment submissions, the interventions of interest included only those treatment regimens used in clinical practice in multiple countries, and the protocol was not registered in a systematic review registry.

**Table 1:** PICOS criteria to identify trials for the systematic literature review

**Population**	Early-stage and locally advanced non-metastatic triple-negative breast cancer
**Interventions**	**Neoadjuvant pembrolizumab regimens:**
	• Pembrolizumab (200 mg q3w × 4 cycles) + carboplatin (AUC 5 q3w × 4 cycles or AUC 1.5 qw × 4 cycles) + paclitaxel (80 mg/ml qw × 4 cycles)
	• Pembrolizumab (200 mg q3w × 4 cycles) + doxorubicin (60 mg/m^2^) or epirubicin (90 mg/ml^2^) + cyclophosphamide (600 mg/m^2^ q3w × 4 cycles)
	• Post-surgery: Pembrolizumab (200 mg q3w × 9 cycles)
	**Preferred neoadjuvant regimens:**
	• Dose-dense doxorubicin + cyclophosphamide followed by paclitaxel every 3 weeks
	• Dose-dense doxorubicin + cyclophosphamide followed by weekly paclitaxel
	• Docetaxel + cyclophosphamide
	**Other neoadjuvant regimens:**
	• Dose-dense doxorubicin + cyclophosphamide
	• Doxorubicin + cyclophosphamide every 3 weeks (category 2B)
	• Cyclophosphamide + methotrexate + fluorouracil
	• Doxorubicin + cyclophosphamide followed by docetaxel every 3 weeks
	• Doxorubicin + cyclophosphamide followed by weekly paclitaxel
	• Epirubicin + cyclophosphamide
	• Docetaxel + doxorubicin + cyclophosphamide
	• Carboplatin + paclitaxel (80 mg/ml qw × 4 cycles)
	• Paclitaxel every 3 weeks followed by dose-dense doxorubicin + cyclophosphamide/ epirubicin/cyclophosphamide
	• Paclitaxel weekly followed by dose-dense doxorubicin + cyclophosphamide
	• Paclitaxel every 3 weeks followed by doxorubicin + cyclophosphamide/ epirubicin/cyclophosphamide
	• Paclitaxel weekly followed by doxorubicin + cyclophosphamide/ epirubicin/cyclophosphamide
	• Nab-paclitaxel followed by (dose-dense) doxorubicin + cyclophosphamide/ epirubicin/cyclophosphamide
	• Nab-paclitaxel + carboplatin followed by (dose-dense) doxorubicin + cyclophosphamide/ epirubicin/cyclophosphamide
	**Neoadjuvant immunotherapy agents:**
	• Atezolizumab + nab-paclitaxel
	• Atezolizumab + nab-paclitaxel followed by atezolizumab + dose-dense doxorubicin + cyclophosphamide
**Comparators**	• Any of the interventions listed above • Any intervention that has been compared to two or more of the above treatments
**Outcomes**	**Efficacy outcomes:**
	• Pathologic complete response (pCR)
	• Event-free survival (EFS)
	• Overall survival (OS)
	• Disease-free survival (DFS)
	• Landmark survival rates
	• Landmark EFS
	• Landmark DFS
	• Treatment duration/time to treatment discontinuation
	**Safety outcomes:**
	• Any adverse events
	• Any grade 3 or higher adverse events
	• Immune-related toxicity
	• Treatment-emergent adverse events (any grade, and grade 3 or higher)
	• Study withdrawals
	**Patient-reported outcomes, including quality of life measures:**
	• EQ-5D
	• EORTC QLQ-C30
	• EORTC QLQ-BR23
	• FACT-B-FBSI
**Time**	Unrestricted
**Study design**	Phase II and III RCTs
	• Parallel group (triple-blind/double-blind)
	• RCT—cross over (triple-blind/double-blind)
	• RCT—post hoc and open-label extension
**Language**	English language

*Abbreviations*: *AUC* Area under the curve, *EQ-5D* European Quality of Life-5D, *EORTC QLQ-C30* European Organisation for Research and Treatment of Cancer Quality-of-Life Questionnaire Core 30, *EORTC QLQ-BR23* European Organisation for Research and Treatment of Cancer Quality of Life Questionnaires-Breast 23, *FACT-B-FBSI* Functional Assessment of Cancer Therapy-Breast-Functional Assessment of Cancer Therapy-Breast Cancer Symptom Index, *RCT* Randomized controlled trial, *TNBC* Triple-negative breast cancer

Relevant trials were identified by searching Excerpta Medica DataBASE (EMBASE), Medical Literature Analysis and Retrieval System Online (MEDLINE), and Cochrane Central Register of Controlled Trials (CENTRAL) through the OVID platform on April 21, 2022 (Additional Tables 1-3). EMBASE and MEDLINE searches were limited to RCTs using the Scottish Intercollegiate Guidelines Network (SIGN) filter (https://www.sign.ac.uk/what-we-do/methodology/search-filters/). Manual searches American Society of Clinical Oncology (2021–2022), European Society of Medical Oncology (2021), and San Antonio Breast Cancer Symposium (2021) conference proceedings were conducted to identify RCTs that had not yet been published in full-text form. In addition, the U.S. National Institute of Health Clinical Trials Registry (clinicaltrials.gov) and European Union Clinical Trial Registry (clinicaltrialsregister.eu) were searched to identify completed RCTs with results available that had not yet been published.

Two reviewers performed abstract selection, full-text selection, and data extraction in duplicate. Any unresolved discrepancies between the two reviewers were resolved by involving a third reviewer and reaching consensus. The Cochrane Collaboration’s Risk of Bias tool was used to assess the risk of bias of included RCTs [13]. 

Following completion of the systematic review, the comparators component of the PICOS was broadened to include any intervention that was compared to two or more interventions of interest, and a targeted literature review was conducted to identify additional RCTs to form a connected network for the NMA.

### Feasibility assessment and network meta-analysis

An extension of pairwise meta-analysis, NMA allows indirect comparisons of interventions that have not been evaluated in head-to-head trials [11]. As the validity of any NMA is based on whether there are systematic differences among trials included in the network across treatment comparisons [11, 14–17], a feasibility assessment was conducted before proceeding with the NMA [14]. 

As only one trial connected each treatment in the networks of evidence, between-study heterogeneity could not be reliably estimated; therefore, NMA was performed with a fixed-effects assumption. The NMA of reported hazard ratios (HRs) in terms of EFS and OS assuming proportional hazards between treatments was performed using a regression model with a contrast-based normal likelihood for the log HR and corresponding standard error of each trial in the network [18]. Normal non-informative prior distributions for the parameters were estimated with a mean of 0 and a variance of 10,000. 

NMA of reported Kaplan–Meier (KM) curves in terms of EFS and OS assuming time-varying hazards between treatment was performed using a fractional polynomial model [11, 19]. Weibull, Gompertz, and second-order fractional polynomial models were considered using a multivariant NMA framework. Reported KM curves were digitized for each treatment arm included in the NMA using DigitizeIt (http://www.digitizeit.xyz). Goodness-of-fit was compared across competing survival models using the deviance information criterion, with a model having a better trade-off between fit and parsimony having a lower deviance information criterion. Relative treatment effects were expressed as odds ratios for pCR and HRs for EFS and OS with 95% credible intervals (CrIs), which reflect a 95% probability that the estimate is within the specified range. Additionally, to allow for time-varying HRs, NMAs with fractional polynomial models representing different survival distributions were fit to the data under a variety of different assumptions about the shape of the hazard function. All analyses were performed using R version 4.0.3 (http://www.r-project.org/) and OpenBugs version 3.2.3 (OpenBUGS Project Management Group) [20].

## Results

### Systematic review

**Fig. 1:**
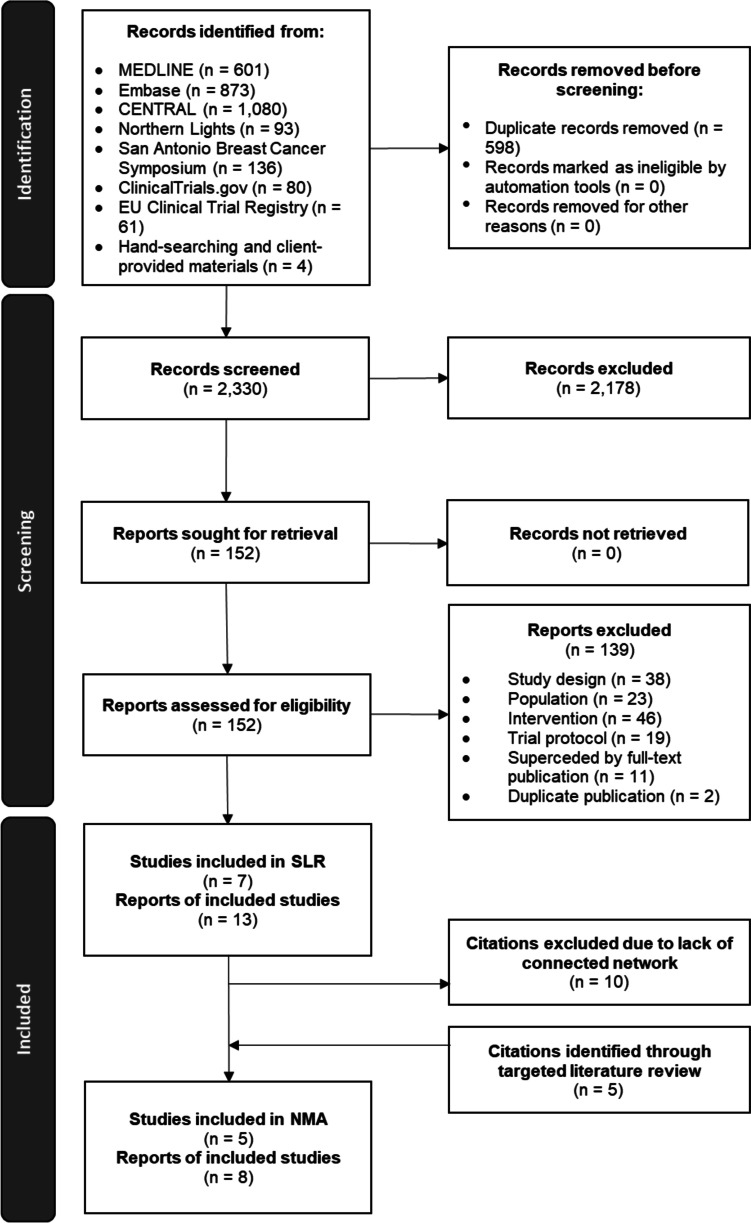
PRISMA flow diagram

The study selection process for identifying trials of interest is outlined in Fig. 1. Thirteen citations pertaining to seven unique trials were identified in the systematic review (Additional Table 4) [6, 8, 21–30]. As the trials identified by the systematic review did not form a connected network of evidence, a targeted literature search was conducted to include any intervention that was compared to two or more treatments of interest, which resulted in the inclusion of five additional citations pertaining to three unique trials. Trials that were not connected in a network, including IMpassion031 evaluating atezolizumab + chemotherapy, were excluded. As a result, a total of eight citations pertaining to five unique trials were included in the feasibility assessment and NMA (Additional Table 5) [22, 23, 31–35].

### Feasibility assessment

Two trials were multinational (KEYNOTE-522 and BrighTNess), two trials were conducted in the U.S. (CALGB 40603 and NeoSTOP), and one trial was conducted in Germany (GeparSepto-GBG 69). KEYNOTE-522 employed quadruple-blind masking, whereas the other trials were open-label (Table 2). All trials exclusively enrolled patients with early-stage, locally advanced non-metastatic TNBC. Patients were enrolled in all trials irrespective of PD-L1 status; however, KEYNOTE-522 measured PD-L1 status using the PD-L1 IHC 22C3 pharmDx test (Dako North America, Inc.) and reported outcomes for PD-L1 subgroups. All trials employed comparable treatment dosing and administration schedules (Additional Table 6).

**Table 2:** Characteristics of trials included in the feasibility assessment and network meta-analysis

Trial ID	Phase	Treatment	Number of patients	Masking	Multicenter	Disease/tumor stage
CALGB 40603[31, 32]	III	Pac + carb➔anthra + cyclo	113	Open-label	Yes	Stage II-III
		Pac + carb + bev➔anthra + cyclo + bev	112			
		Pac + bev➔anthra + cyclo + bev	110			
		Pac➔anthra + cyclo	108			
BrighTNess[34, 35]	III	Pac + carb➔anthra + cyclo	160	Open-label	Yes	Clinical stage T2-3 N0-2 or T1 N1-2
		Pac➔anthra + cyclo	158			
		Pac + carb + veli➔anthra + cyclo	316			
GeparSepto-GBG 69[22, 23]	III	Pac➔anthra + cyclo	606	Open-label	Yes	cT2—cT4a-d, cT1c and cN + , cT1c and pNSLN + , cT1c and ER-negative and PR-negative, or cT1c and Ki67 > 20%, or cT1c and HER2-positive
		Nab-pac➔anthra + cyclo	606			
KEYNOTE-522	III	Pembro + pac + carb➔pembro + anthra + cyclo➔adjuvant pembro	784	Quadruple-blind	Yes	Stage II-III
		Pac + carb➔anthra + cyclo	390			
NeoSTOP[33]	II	Pac + carb➔anthra + cyclo	48	Open-label	Yes	Stage I-III
		Doc + carb➔anthra + cyclo	52			

*Abbreviations*: *Anthra* Anthracycline, *bev* Bevacizumab, *carb* Carboplatin; *cyclo* Cyclophosphamide, *doc* Docetaxel, *dox* Doxorubicin, *nab-pac* Nab-paclitaxel, *pac* Paclitaxel, *pembro* Pembrolizumab, *veli* Veliparib

Arrows (➔) indicate where treatment was administered sequentially; treatments to the left of the arrow were administered first. Anthra includes dox and epi, which were assumed to be equivalent

Although baseline patient characteristics were not reported for most trials, the available data on patient characteristics and enrollment criteria suggest no important between-trial differences (Additional Table 7). Among the trials reporting baseline patient characteristics, median age ranged from 48 to 54 years, 99.9–100% of patients were female, and 69–74% of patients were Caucasian. Based on trial eligibility criteria, four trials enrolled patients with an ECOG performance score of 0 or 1, whereas one trial (GeparSepto-GBG 69) enrolled patients with a Karnofsky performance score of 70–80% or better. 

All five trials reported pCR. Survival outcomes for CALGB 40603 were only available from the US National Institutes of Health Clinical Trial Registry, which reported identical HRs and 95% confidence intervals for both OS and recurrence-free survival (which was defined similarly as EFS in the other included trials, Additional Table 8). As this appeared to be a reporting error, CALGB 40603 was excluded from the primary NMA of OS and EFS; sensitivity analyses including this trial are reported in Additional Fig. 4 and Additional Tables 19–20. In addition, as GeparSepto-GBG 69 did not provide KM curves for OS, this trial was only included in the constant HR NMA model for OS. Data sources for each outcome measure are presented in Additional Table 9.

### Network meta-analysis

**Fig. 2:**
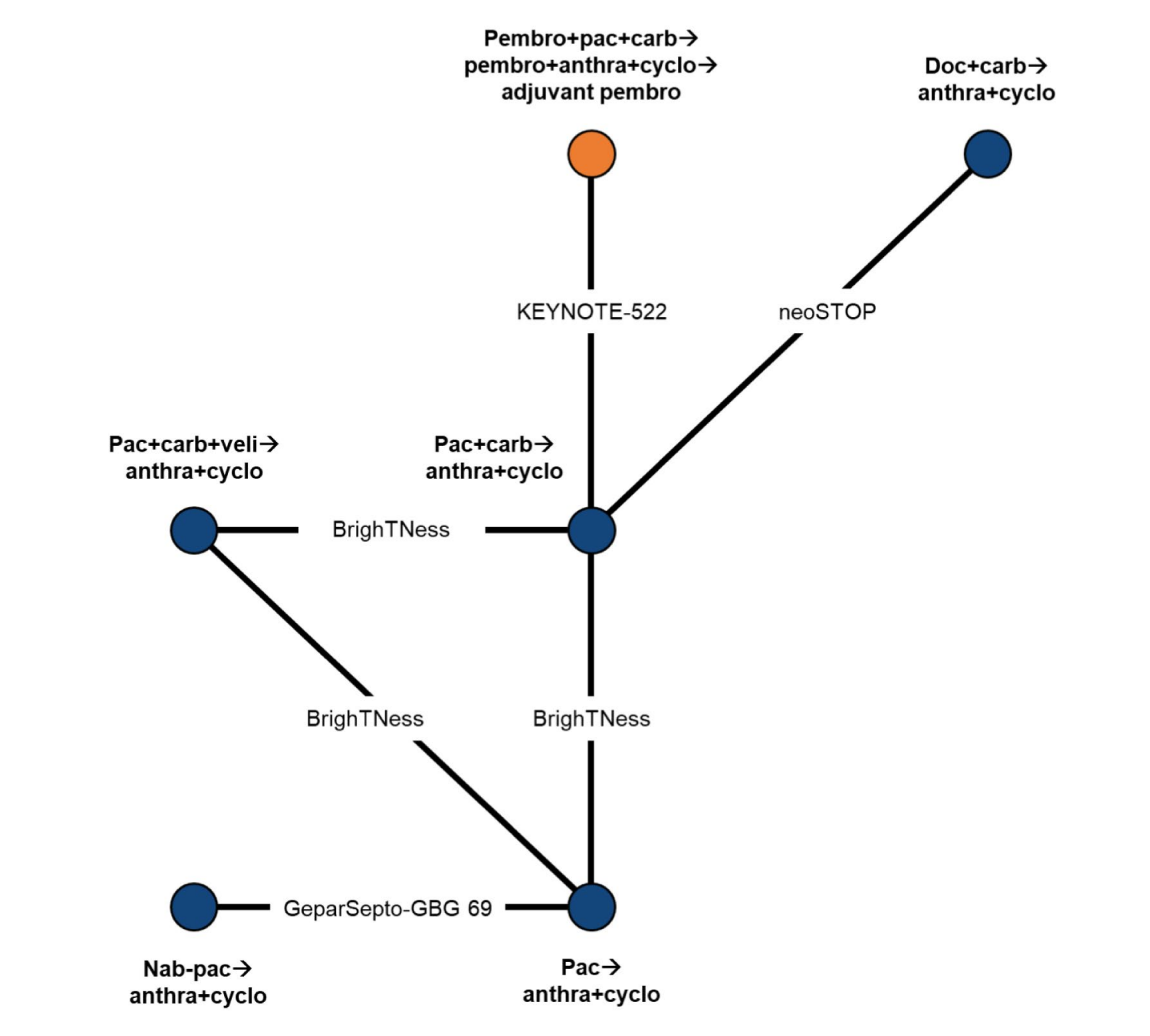
Network of evidence for pathologic complete response. Arrows (→) indicate where treatment was administered sequentially, with treatments to the left of the arrow administered first. The orange circle denotes the primary treatment regimen of interest. Anthra includes doxorubicin and epirubicin, which were assumed to be equivalent. Anthra = anthracycline; bev = bevacizumab; carb = carboplatin; cyclo = cyclophosphamide; doc = docetaxel; nab-pac = nab-paclitaxel; pac = paclitaxel; pembro = pembrolizumab; veli = veliparib

**Fig. 3:**
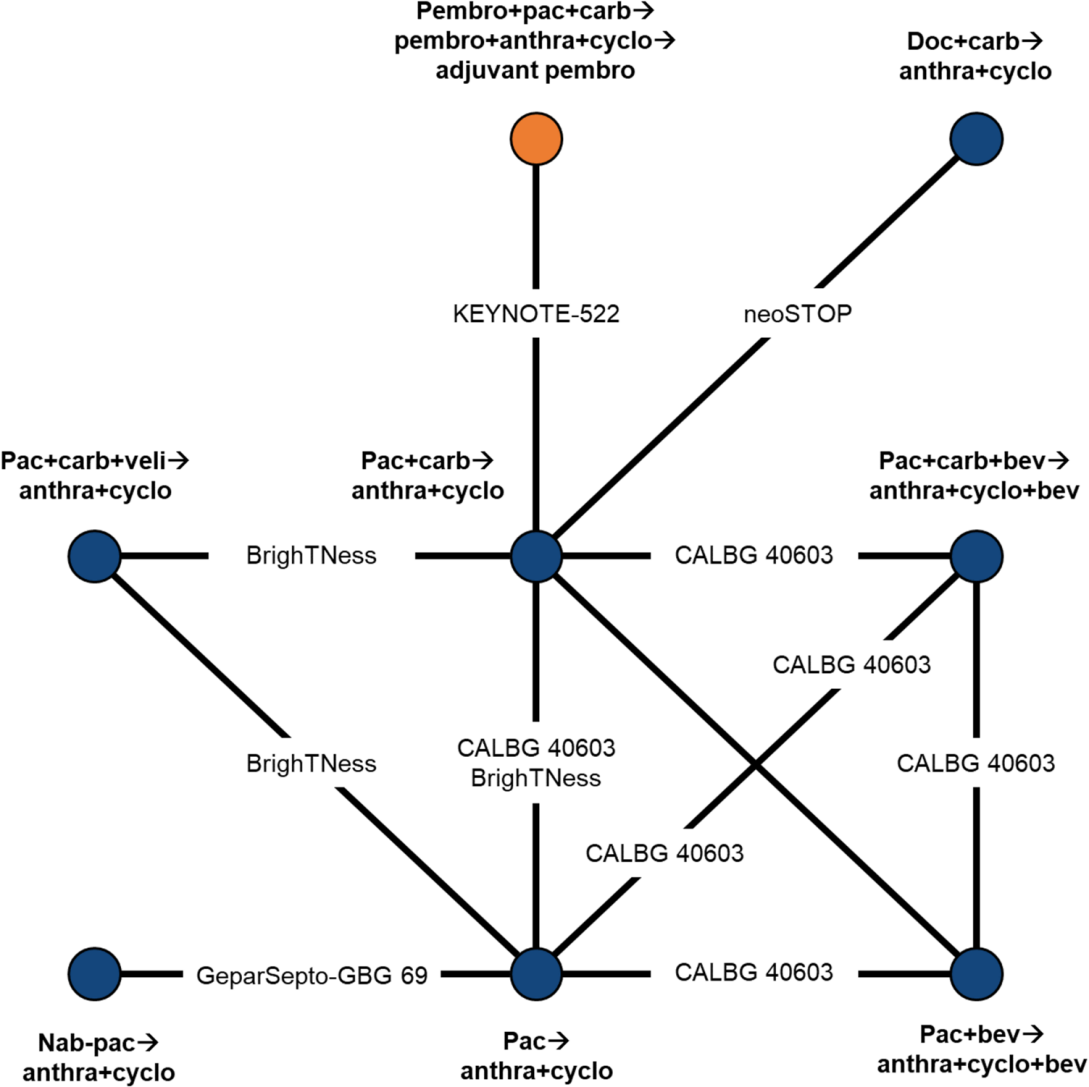
Network of evidence for event-free survival and overall survival. Arrows (→) indicate where treatment was administered sequentially, with treatments to the left of the arrow administered first. The orange circle denotes the primary treatment regimen of interest. Anthra includes doxorubicin and epirubicin, which were assumed to be equivalent. Anthra = anthracycline; bev = bevacizumab; carb = carboplatin; cyclo = cyclophosphamide; doc = docetaxel; nab-pac = nab-paclitaxel; pac = paclitaxel; pembro = pembrolizumab; veli = veliparib

Networks were constructed to compare neoadjuvant pembrolizumab + chemotherapy followed by adjuvant pembrolizumab to other neoadjuvant treatment regimens. A fundamental assumption was made in the inclusion of trials evaluating chemotherapy regimens without analyses by PD-L1 subgroups: PD-L1 expression levels only influenced the PD-L1-directed therapy-containing regimen (i.e., pembrolizumab). Also, as doxorubicin and epirubicin have similar efficacy profiles [36], networks of evidence were constructed by treating cohorts assigned to doxorubicin or epirubicin as equivalent (classified as anthracycline). Five trials were included in the pCR network (Fig. 2), and four trials were included in the OS and EFS networks (Fig. 3).

For pCR, neoadjuvant pembrolizumab + paclitaxel + carboplatin followed by pembrolizumab + anthracycline + cyclophosphamide followed by adjuvant pembrolizumab showed statistically favorable improvements in pCR versus paclitaxel + carboplatin followed by anthracycline + cyclophosphamide, paclitaxel followed by anthracycline + cyclophosphamide, and paclitaxel + bevacizumab followed by anthracycline + cyclophosphamide + bevacizumab (Table 3).

**Table 3:** Results of network meta-analysis for pathologic complete response

**Intervention**	Pac + carb➔anthra + cyclo	Pac➔anthra + cyclo	Doc + carb➔anthra + cyclo	Nab-pac➔anthra + cyclo	Pac + bev➔anthra + cyclo + bev	Pac + carb + bev➔anthra + cyclo + bev	Pac + carb + vel➔anthra + cyclo	Pembro + pac + carb➔pembro + anthra + cyclo➔adjuvant pembro
Pac + carb➔anthra + cyclo	1	–	–	–	–	–	–	–
Pac➔anthra + cyclo	0.43 (0.30, 0.61)	1	–	–	–	–	–	–
Doc + carb➔anthra + cyclo	0.99 (0.45, 2.16)	2.27 (0.97, 5.37)	1	–	–	–	–	–
Nab-pac➔anthra + cyclo	1.13 (0.62, 2.13)	**2.61 (1.58, 4.41)**	1.16 (0.42, 3.12)	1	–	–	–	–
Pac + bev➔anthra + cyclo + bev	0.72 (0.43, 1.19)	1.65 (0.99, 2.71)	0.73 (0.29, 1.84)	0.63 (0.30, 1.28)	1	–	–	–
Pac + carb + bev➔anthra + cyclo + bev	1.51 (0.90, 2.55)	**3.47 (2.08, 5.93)**	1.54 (0.61, 3.94)	1.33 (0.63, 2.76)	**2.12 (1.21, 3.68)**	1	–	–
Pac + carb + vel➔anthra + cyclo	0.95 (0.67, 1.36)	**2.19 (1.53, 3.17)**	0.96 (0.41, 2.28)	0.84 (0.44, 1.56)	1.33 (0.75, 2.34)	0.63 (0.35, 1.13)	1	–
Pembro + pac + carb➔pembro + anthra + cyclo➔adjuvant pembro	**1.36 (1.06, 1.73)**	**3.12 (2.04, 4.85)**	1.37 (0.61, 3.14)	1.19 (0.61, 2.30)	**1.89 (1.07, 3.30)**	0.89 (0.50, 1.59)	1.42 (0.92, 2.21)	1

*Abbreviations*: *Anthra* Anthracycline, *bev* bevacizumab, *carb* Carboplatin, *cyclo* Cyclophosphamide, *doc* Docetaxel, *nab-pac* Nab-paclitaxel, *pac* Paclitaxel, *pembro* Pembrolizumab, *veli* Veliparib

Each cell represents the comparison (odds ratio and 95% CrI) of the row treatment versus the column treatment. All bolded values are statistically meaningful at the 0.05 significance level. Deviance information criterion: 26.59; Deviance: 14.65

For EFS, neoadjuvant pembrolizumab + paclitaxel + carboplatin followed by pembrolizumab + anthracycline + cyclophosphamide followed by adjuvant pembrolizumab showed statistically favorable improvements in EFS versus paclitaxel followed by anthracycline + cyclophosphamide, paclitaxel + carboplatin followed by anthracycline + cyclophosphamide, and paclitaxel + carboplatin + veliparib followed by anthracycline + cyclophosphamide (Table 4).

**Table 4:** Results of network meta-analysis for event-free survival

**Intervention**	Pac➔anthra + cyclo	Pac + carb➔anthra + cyclo	Doc + carb➔anthra + cyclo	Nab-pac➔anthra + cyclo	Pac + carb + veli➔anthra + cyclo	Pembro + pac + carb➔pembro + anthra + cyclo➔adjuvant pembro
Pac➔anthra + cyclo	1	–	–	–	–	–
Pac + carb➔anthra + cyclo	**0.57 (0.36, 0.90)**	1	–	–	–	–
Doc + carb➔anthra + cyclo	0.66 (0.17, 2.59)	1.16 (0.32, 4.26)	1	–	–	–
Nab-pac➔anthra + cyclo	**0.62 (0.39, 0.99)**	1.09 (0.56, 2.13)	0.94 (0.22, 4.06)	1	–	–
Pac + carb + veli➔anthra + cyclo	**0.63 (0.43, 0.93)**	1.10 (0.71, 1.71)	0.95 (0.24, 3.77)	1.01 (0.56, 1.88)	1	–
Pembro + pac + carb➔pembro + anthra + cyclo➔adjuvant pembro	**0.36 (0.21, 0.61)**	**0.63 (0.48, 0.82)**	0.54 (0.14, 2.09)	0.58 (0.28, 1.17)	**0.57 (0.34, 0.95)**	1

*Abbreviations*: *Anthra* Anthracycline, *carb* Carboplatin, *CrI* Credible Interval, *cyclo* Cyclophosphamide, *doc* Docetaxel, *HR* Hazard ratio, *nab-pac* nab-paclitaxel, *pac* Paclitaxel, *pembro* Pembrolizumab, *veli* Veliparib

Each cell represents the comparison (HR and 95% CrI) of the row treatment versus the column treatment. All bolded values are statistically meaningful at the 0.05 significance level. Deviance information criterion: 9.23; Deviance: 4.26

For OS, neoadjuvant pembrolizumab + paclitaxel + carboplatin followed by pembrolizumab + anthracycline + cyclophosphamide followed by adjuvant pembrolizumab showed statistically favorable OS results versus paclitaxel followed by anthracycline + cyclophosphamide (Table 5).

**Table 5:** Results of network meta-analysis for overall survival

**Intervention**	Pac➔anthra + cyclo	Pac + carb➔anthra + cyclo	Doc + carb➔anthra + cyclo	Nab-pac➔anthra + cyclo	Pac + carb + veli➔anthra + cyclo	Pembro + pac + carb➔pembro + anthra + cyclo➔adjuvant pembro
Pac➔anthra + cyclo	1	–	–	–	–	–
Pac + carb➔anthra + cyclo	0.63 (0.33, 1.20)	1	–	–	–	–
Doc + carb➔anthra + cyclo	0.82 (0.16, 4.18)	1.30 (0.29, 5.87)	1	–	–	–
Nab-pac➔anthra + cyclo	0.74 (0.40, 1.38)	1.18 (0.48, 2.86)	0.91 (0.16, 5.32)	1	–	–
Pac + carb + veli➔anthra + cyclo	0.82 (0.48, 1.39)	1.30 (0.72, 2.35)	1.00 (0.20, 4.94)	1.11 (0.50, 2.54)	1	–
Pembro + pac + carb➔pembro + anthra + cyclo➔adjuvant pembro	**0.45 (0.22, 0.95)**	0.72 (0.51, 1.02)	0.55 (0.12, 2.59)	0.61 (0.23, 1.60)	0.55 (0.28, 1.09)	1

*Abbreviations*: *Anthra* Anthracycline, *Carb* Carboplatin, *Cyclo* Cyclophosphamide; *CrI* Credible interval, *DIC* Deviance information criterion, *Doc* Docetaxel, *HR* Hazard ratio, *Nab-pac* Nab-paclitaxel, *Pac* Paclitaxel, *Pembro* Pembrolizumab, *Veli* Veliparib

Each cell represents the comparison (HR and 95% CrI) of the row treatment versus the column treatment

All bolded values are statistically meaningful at the 0.05 significance level. DIC: 9.25; Deviance: 4.26

As there were no major violations of the assumption that HRs were proportional over time (determined by log–log and Schoenfeld residual plots), the best-fitting models were determined to be constant HR models. Further, the best-fitting time-varying models for both EFS and OS did not show statistically meaningful changes in HR over time for any treatment (Additional Figs. 1-3, Additional Tables 10–17). Therefore, the constant HR results provided the best combination of fit and parsimony for all treatments.

## Discussion

NMA including five RCTs of neoadjuvant immunotherapy or chemotherapy regimens for patients with early-stage or locally advanced, non-metastatic TNBC demonstrated that neoadjuvant pembrolizumab + chemotherapy followed by adjuvant pembrolizumab had more favorable treatment outcomes than certain other neoadjuvant treatment regimens. In particular, the relative efficacy of neoadjuvant pembrolizumab + chemotherapy followed by adjuvant pembrolizumab was statistically favorable to paclitaxel followed by anthracycline + cyclophosphamide in terms of pCR, EFS, and OS; to paclitaxel + carboplatin followed by anthracycline + cyclophosphamide in terms of pCR and EFS; to paclitaxel + bevacizumab followed by anthracycline + cyclophosphamide + bevacizumab in terms of pCR; and to paclitaxel + carboplatin + veliparib followed by anthracycline + cyclophosphamide in terms of EFS. Thus, neoadjuvant pembrolizumab + chemotherapy followed by adjuvant pembrolizumab may be more efficacious than other neoadjuvant treatment regimens for patients with high-risk, early-stage TNBC.

The validity of NMA findings are dependent on the quality of the individual RCTs included in the network and the degree to which these RCTs are similar in terms of populations and methodology [9–11]. In an NMA of RCTs involving multiple treatment comparisons, randomization holds only within individual RCTs and not across RCTs. Thus, if the direct comparisons between treatments in the network involve systematic between-trial differences in study or patient characteristics, and these differences are treatment effect modifiers, then the estimates of indirect comparisons will be biased. In this study, the feasibility assessment demonstrated that the distribution of most potential treatment effect modifiers was balanced across the RCTs. However, KEYNOTE-522 was the only trial that evaluated a treatment regimen spanning both the neoadjuvant and adjuvant phases, which may have served to influence the relative treatment effects.

Some factors limit the conclusions that can be drawn from this NMA. First, ambiguity in the reporting of recurrence-free survival and OS for CALGB 40603 precluded the inclusion of this trial in the survival analyses. Second, because only one study connected each treatment in the network of evidence, between-study heterogeneity could not be estimated, and the NMA was performed with a fixed-effects assumption. Third, the systematic review did not identify sufficient data to answer questions such as which biomarkers predict pCR, which patients may achieve pCR without pembrolizumab, or the ideal dosage of pembrolizumab to achieve an ideal risk/benefit ratio, which could be investigated in future studies.

Despite these limitations, this study has several strengths that maximize its comprehensiveness and rigor. In particular, highly sensitive systematic searches in the peer-reviewed literature, recent conferences, and clinical trial registries were employed to identify all published evidence from RCTs of neoadjuvant immunotherapy and chemotherapy treatments for early-stage, locally advanced, non-metastatic TNBC. In addition, the review process was guided by pre-defined eligibility criteria, and data quality was ensured through the involvement of two independent reviewers in the study selection and data extraction processes. 

## Conclusions

The results of this systematic review and NMA suggest that neoadjuvant pembrolizumab + chemotherapy followed by adjuvant pembrolizumab is an effective treatment compared with other neoadjuvant treatments for patients with previously untreated, locally advanced, non-metastatic TNBC.

### Supplementary Information

**Additional file 1: Additional Table 1.** PRISMA 2020 checklist. **Additional Table 2.** Search strategy for EMBASE 1974 to 2022 April 20.**Additional Table 3.** Search strategy for Ovid MEDLINE(R) In-Process & Other Non-Indexed Citations, Ovid MEDLINE(R) Daily and Ovid MEDLINE(R) 1946 to April 20, 2021.**Additional Table 4.** Search strategy for EBM Reviews - Cochrane Central Register of Controlled Trials March 2022. **Additional Table 5.** Trials identified in the systematic review.**Additional Table 6.** Trials included in the feasibility assessment and network meta-analysis.**Additional Table 7.** Treatment characteristics of included trials.** Supplementary Table 8.** Baseline patient characteristics of included trials.**Additional Table 9.** Definitions of pathological complete response, overall survival, and event-free survival used in the included trials.**Additional Table 10.** Data sources for feasibility assessment and network meta-analysis**. Additional Figure 1.** Results of network meta-analysis for event-free survival based on time-varying hazard ratios (constant hazards with p_1_=0.5, p_2_=0).** Additional Figure 2.** Best-fitting model: Results of network meta-analysis for overall survival based on constant hazard ratio model with p_1_=0, p_2_=0.**Additional Figure 3.** Second best-fitting model: Results of network meta-analysis for overall survival based on second-order fractional polynomial model with p_1_=0, p_2_=0.5; scale and second shape.** Additional Table 11.** Model fit estimate for network meta-analysis for event-free survival with parametric survival models.** Additional Table 12.** Estimated hazard ratios for event-free survival versus paclitaxel followed by anthracycline + cyclophosphamide at select time points based on time-varying hazard ratio assumption (constant hazard ratio with p_1_=0.5, p_2_=0).** Additional Table 13.** Basic parameter estimates of constant hazard ratio model with p_1_=0.5, p_2_=0 for event-free survival.** Additional Table 14.** Model fit estimate for network meta-analysis for overall survival with parametric survival models.** Additional Table 15.** Best-fitting model for overall survival: Estimated hazard ratios versus paclitaxel followed by anthracycline + cyclophosphamide at select time points based on time-varying hazard ratio assumption (constant hazard ratio with p_1_=0, p_2_=0). **Additional Table 16.** Best-fitting model for overall survival: Basic parameter estimates of constant hazard ratio model with p_1_=0, p_2_=0.** Additional Table 17.** Second best-fitting model for overall survival: Estimated hazard ratios versus paclitaxel followed by anthracycline + cyclophosphamide at select time points based on time-varying hazard ratio assumption (second-order fractional polynomial with p_1_=0, p_2_=0.5).** Additional Table 18.** Second best-fitting model for overall survival: Basic parameter estimates of second-order polynomial model with p_1_=0, p_2_=0.5. **Additional Figure 4.** Network of evidence for event-free survival and overall survival including CALGB 40603 (Alliance). Arrows (➔) indicate where treatment was administered sequentially, with treatments to the left of the arrow administered first. The orange circle denotes the primary treatment regimen of interest. Anthra includes doxorubicin and epirubicin, which were assumed to be equivalent. Anthra = anthracycline; bev = bevacizumab; carb = carboplatin; cyclo = cyclophosphamide; doc = docetaxel; nab-pac = nab-paclitaxel; pac = paclitaxel; pembro = pembrolizumab; veli = veliparib. **Additional Table 19.** Results of network-analysis for event-free survival including CALGB 40603 (Alliance).** Additional Table 20.** Results of network-analysis for event-free survival including CALGB 40603 (Alliance).

## Data Availability

The datasets analyzed in this study are available from the corresponding author on reasonable request.

## Abbreviations

CENTRAL: Cochrane Central Register of Controlled Trials
EFS: Event-free survival
EMBASE: Excerpta Medica DataBASE
HR: Hazard ratio
KM: Kaplan-Meier
MEDLINE: Medical Literature Analysis and Retrieval System Online
NMA: Network meta-analysis
OS: Overall survival
pCR: Pathologic complete response
PD-1: Programmed cell death protein 1
PD-L1: Programmed cell death ligand 1
PICOS: Population, intervention, comparator, outcomes, study design
PFS: Progression-free survival
PRISMA: Preferred Reporting Items for Systematic Reviews and Meta-analysis
RCT: Randomized controlled trial
SIGN: Scottish Intercollegiate Guidelines Network
TNBC: Triple-negative breast cancer

## References

[1] Sung H, Ferlay J, Siegel RL, Laversanne M, Soerjomataram I, Jemal A, Bray F (2021). Global Cancer Statistics 2020: GLOBOCAN Estimates of Incidence and Mortality Worldwide for 36 Cancers in 185 Countries. CA Cancer J Clin.

[2] Yao H, He G, Yan S, Chen C, Song L, Rosol TJ, Deng X (2017). Triple-negative breast cancer: is there a treatment on the horizon?. Oncotarget.

[3] Bou Zerdan M, Ghorayeb T, Saliba F, Allam S, Bou Zerdan M, Yaghi M, Bilani N, Jaafar R, Nahleh Z: Triple negative breast cancer: updates on classification and treatment in 2021. Cancers 2022, 14(5). 10.3390/cancers14051253 PMC8909187 35267561

[4] Mayer IA, Abramson VG, Lehmann BD, Pietenpol JA (2014). New strategies for triple-negative breast cancer—deciphering the heterogeneity. Clin Cancer Res.

[5] Yin L, Duan J-J, Bian X-W (2020). Yu S-c: Triple-negative breast cancer molecular subtyping and treatment progress. Breast Cancer Res.

[6] Schmid P, Cortes J, Pusztai L, McArthur H, Kümmel S, Bergh J, Denkert C, Park YH, Hui R, Harbeck N (2020). Pembrolizumab for Early Triple-Negative Breast Cancer. N Engl J Med.

[7] FDA approves pembrolizumab for high-risk early-stage triple-negative breast cancer. https://www.fda.gov/drugs/resources-information-approved-drugs/fda-approves-pembrolizumab-high-risk-early-stage-triple-negative-breast-cancer .

[8] Mittendorf EA, Zhang H, Barrios CH, Saji S, Jung KH, Hegg R, Koehler A, Sohn J, Iwata H, Telli ML (2020). Neoadjuvant atezolizumab in combination with sequential nab-paclitaxel and anthracycline-based chemotherapy versus placebo and chemotherapy in patients with early-stage triple-negative breast cancer (IMpassion031): a randomised, double-blind, phase 3 trial. Lancet.

[9] Mills EJ, Thorlund K, Ioannidis JPA (2013). Demystifying trial networks and network meta-analysis. BMJ.

[10] Mills EJ, Ioannidis JP, Thorlund K, Schünemann HJ, Puhan MA, Guyatt GH (2012). How to use an article reporting a multiple treatment comparison meta-analysis. JAMA.

[11] Jansen JP, Naci H (2013). Is network meta-analysis as valid as standard pairwise meta-analysis? It all depends on the distribution of effect modifiers. BMC Med.

[12] Moher D, Liberati A, Tetzlaff J, Altman DG, P Group (2009). Preferred reporting items for systematic reviews and meta-analyses: the PRISMA statement. J Clin Epidemiol.

[13] Higgins JP, Thomas J, Chandler J, Cumpston M, Li T, Page MJ, Welch VA: Cochrane handbook for systematic reviews of interventions: John Wiley & Sons; 2019. 10.1002/14651858.ED000142 PMC10284251 31643080

[14] Cope S, Zhang J, Saletan S, Smiechowski B, Jansen JP, Schmid P (2014). A process for assessing the feasibility of a network meta-analysis: a case study of everolimus in combination with hormonal therapy versus chemotherapy for advanced breast cancer. BMC Med.

[15] Cooper NJ, Sutton AJ, Morris D, Ades AE, Welton NJ (2009). Addressing between-study heterogeneity and inconsistency in mixed treatment comparisons: application to stroke prevention treatments in individuals with non-rheumatic atrial fibrillation. Stat Med.

[16] Dias S, Sutton AJ, Ades AE, Welton NJ (2013). Evidence synthesis for decision making 2: a generalized linear modeling framework for pairwise and network meta-analysis of randomized controlled trials. Med Decis Mak.

[17] Jansen JP, Fleurence R, Devine B, Itzler R, Barrett A, Hawkins N, Lee K, Boersma C, Annemans L, Cappelleri JC (2011). Interpreting indirect treatment comparisons and network meta-analysis for health-care decision making: report of the ISPOR Task Force on Indirect Treatment Comparisons Good Research Practices: part 1. Value Health.

[18] Dias S, Welton NJ, Sutton AJ, Ades A (2011). NICE DSU technical support document 2: a generalised linear modelling framework for pairwise and network meta-analysis of randomised controlled trials.

[19] Jansen JP, Cope S (2012). Meta-regression models to address heterogeneity and inconsistency in network meta-analysis of survival outcomes. BMC Med Res Methodol.

[20] R Development Core Team (2020). R: A language and environment for statistical computing. R Foundation for Statistical Computing.

[21] Gianni L, Mansutti M, Anton A, Calvo L, Bisagni G, Bermejo B, Semiglazov V, Thill M, Chacon JI, Chan A (2018). Comparing Neoadjuvant Nab-paclitaxel vs Paclitaxel Both Followed by Anthracycline Regimens in Women With ERBB2/HER2-Negative Breast Cancer-The Evaluating Treatment With Neoadjuvant Abraxane (ETNA) Trial: A Randomized Phase 3 Clinical Trial. JAMA Oncol.

[22] Untch M, Jackisch C, Schneeweiss A, Conrad B, Aktas B, Denkert C, Eidtmann H, Wiebringhaus H, Kümmel S, Hilfrich J (2016). Nab-paclitaxel versus solvent-based paclitaxel in neoadjuvant chemotherapy for early breast cancer (GeparSepto-GBG 69): a randomised, phase 3 trial. Lancet Oncol.

[23] Untch M, Jackisch C, Schneeweiss A, Schmatloch S, Aktas B, Denkert C, Schem C, Wiebringhaus H, Kümmel S, Warm M (2019). NAB-Paclitaxel Improves Disease-Free Survival in Early Breast Cancer: GBG 69–GeparSepto. J Clin Oncol.

[24] Mittendorf E, Harbeck N, Zhang H, Saji S, Jung KH, Patel S, Patel S, Duc AN, Liste-Hermoso M, Chui SY (2021). Abstract PD12–11: Patient-reported outcomes from the Phase III IMpassion031 trial of neoadjuvant atezolizumab + chemotherapy in early triple-negative breast cancer. Cancer Research.

[25] Dent R, Cortes J, Pusztai L, McArthur HL, Kuemmel S, Bergh J, Denkert C, Park YH, Hui R, Harbeck N (2020). 1O KEYNOTE-522 Asian subgroup: phase III study of neoadjuvant pembrolizumab (pembro) vs placebo (pbo) + chemotherapy (chemo) followed by adjuvant pembro vs pbo for early triple-negative breast cancer (TNBC). Ann Oncol.

[26] Chen X, Ye G, Zhang C, Li X, Chen Y, Xie X, Zheng H, Cao Y, Wu K, Ni D (2013). Superior outcome after neoadjuvant chemotherapy with docetaxel, anthracycline, and cyclophosphamide versus docetaxel plus cyclophosphamide: results from the NATT trial in triple negative or HER2 positive breast cancer. Breast Cancer Res Treat.

[27] Chen X, Ye G, Zhang C, Li X, Shen K (2016). Non-anthracycline-containing docetaxel and cyclophosphamide regimen is associated with sustained worse outcome compared with docetaxel, anthracycline and cyclophosphamide in neoadjuvant treatment of triple negative and HER2-positive breast cancer patients: updated follow-up data from NATT study. Chin J Cancer Res.

[28] Ademuyiwa FO, Gao F, Chen I, Northfelt DW, Wesolowski R, Arora M, Brufsky A, Dees C, Santa-Maria CA, Connolly RM et al: Nci 10013 - A randomized phase 2study of neoadjuvant carboplatin and paclitaxel, with or without atezolizumab in triple negative breast cancer(TNBC). Cancer Res 2021, 81(4 SUPPL). 10.1038/s41523-022-00500-3 PMC9803651 36585404

[29] Vriens BE, Aarts MJ, de Vries B, van Gastel SM, Wals J, Smilde TJ, van Warmerdam LJ, de Boer M, van Spronsen DJ, Borm GF (2013). Doxorubicin/cyclophosphamide with concurrent versus sequential docetaxel as neoadjuvant treatment in patients with breast cancer. Eur J Cancer.

[30] Schmid P, Cortes J, Dent R, Pusztai L, McArthur H, Kümmel S, Bergh J, Denkert C, Park YH, Hui R (2022). Event-free Survival with Pembrolizumab in Early Triple-Negative Breast Cancer. N Engl J Med.

[31] Paclitaxel With or Without Carboplatin and/or Bevacizumab Followed by Doxorubicin and Cyclophosphamide in Treating Patients With Breast Cancer That Can Be Removed by Surgery. https://clinicaltrials.gov/ct2/show/NCT00861705.

[32] Sikov WM, Berry DA, Perou CM, Singh B, Cirrincione CT, Tolaney SM, Kuzma CS, Pluard TJ, Somlo G, Port ER (2015). Impact of the addition of carboplatin and/or bevacizumab to neoadjuvant once-per-week paclitaxel followed by dose-dense doxorubicin and cyclophosphamide on pathologic complete response rates in stage II to III triple-negative breast cancer: CALGB 40603 (Alliance). J Clin Oncol.

[33] Sharma P, Kimler BF, O'Dea A, Nye L, Wang YY, Yoder R, Staley JM, Prochaska L, Wagner J, Amin AL (2021). Randomized Phase II Trial of Anthracycline-free and Anthracycline-containing Neoadjuvant Carboplatin Chemotherapy Regimens in Stage I-III Triple-negative Breast Cancer (NeoSTOP). Clin Cancer Res.

[34] Loibl S, Sikov W, Huober J, Rugo H, Wolmark N, O'Shaughnessy J, Maag D, Untch M, Golshan M, Lorenzo JP (2021). 119O Event-free survival (EFS), overall survival (OS), and safety of adding veliparib (V) plus carboplatin (Cb) or carboplatin alone to neoadjuvant chemotherapy in triple-negative breast cancer (TNBC) after≥ 4 years of follow-up: BrighTNess, a randomized phase III trial. Ann Oncol.

[35] Loibl S, O'Shaughnessy J, Untch M, Sikov WM, Rugo HS, McKee MD, Huober J, Golshan M, von Minckwitz G, Maag D (2018). Addition of the PARP inhibitor veliparib plus carboplatin or carboplatin alone to standard neoadjuvant chemotherapy in triple-negative breast cancer (BrighTNess): a randomised, phase 3 trial. Lancet Oncol.

[36] Khasraw M, Bell R, Dang C (2012). Epirubicin: Is it like doxorubicin in breast cancer? A clinical review. Breast.

