# Supplementary material for: Neoadjuvant immunotherapy and chemotherapy regimens for the treatment of high-risk, early-stage triple-negative breast cancer: a systematic review and network meta-analysis
Source: BMC Cancer. 2023 Aug 23;23:792. doi: 10.1186/s12885-023-11293-4 (PMC10463750; doi:10.1186/s12885-023-11293-4)
Supplement: Supplementary file 1 — Additional file 1: Additional Table 1. PRISMA 2020 checklist. Additional Table 2. Search strategy for EMBASE 1974 to 2022 April 20.Additional Table 3. Search strategy for Ovid MEDLINE(R) In-Process & Other Non-Indexed Citations, Ovid MEDLINE(R) Daily and Ovid MEDLINE(R) 1946 to April 20, 2021.Additional Table 4. Search strategy for EBM Reviews - Cochrane Central Register of Controlled Trials March 2022. Additional Table 5. Trials identified in the systematic review.Additional Table 6. Trials included in the feasibility assessment and network meta-analysis.Additional Table 7. Treatment characteristics of included trials. Supplementary Table 8. Baseline patient characteristics of included trials.Additional Table 9. Definitions of pathological complete response, overall survival, and event-free survival used in the included trials.Additional Table 10. Data sources for feasibility assessment and network meta-analysis. Additional Figure 1. Results of network meta-analysis for event-free survival based on time-varying hazard ratios (constant hazards with p1=0.5, p2=0). Additional Figure 2. Best-fitting model: Results of network meta-analysis for overall survival based on constant hazard ratio model with p1=0, p2=0.Additional Figure 3. Second best-fitting model: Results of network meta-analysis for overall survival based on second-order fractional polynomial model with p1=0, p2=0.5; scale and second shape. Additional Table 11. Model fit estimate for network meta-analysis for event-free survival with parametric survival models. Additional Table 12. Estimated hazard ratios for event-free survival versus paclitaxel followed by anthracycline + cyclophosphamide at select time points based on time-varying hazard ratio assumption (constant hazard ratio with p1=0.5, p2=0). Additional Table 13. Basic parameter estimates of constant hazard ratio model with p1=0.5, p2=0 for event-free survival. Additional Table 14. Model fit estimate for network meta-analysis for overall survival wit [file 12885_2023_11293_MOESM1_ESM.docx]

**Neoadjuvant immunotherapy and chemotherapy regimens for the treatment of high-risk, early-stage triple-negative breast cancer: A systematic review and network meta-analysis**

ADDITIONAL MATERIAL

Additional Table 1. PRISMA 2020 checklist

| **Section and Topic** | **Item #** | **Checklist item** | **Location where item is reported** |
| --- | --- | --- | --- |
| **TITLE** | | |  |
| Title | 1 | Identify the report as a systematic review. | Page 1 |
| **ABSTRACT** | | |  |
| Abstract | 2 | See the PRISMA 2020 for Abstracts checklist. | Pages 1-2 |
| **INTRODUCTION** | | |  |
| Rationale | 3 | Describe the rationale for the review in the context of existing knowledge. | Page 4 |
| Objectives | 4 | Provide an explicit statement of the objective(s) or question(s) the review addresses. | Page 4 |
| **METHODS** | | |  |
| Eligibility criteria | 5 | Specify the inclusion and exclusion criteria for the review and how studies were grouped for the syntheses. | Table 1 |
| Information sources | 6 | Specify all databases, registers, websites, organisations, reference lists and other sources searched or consulted to identify studies. Specify the date when each source was last searched or consulted. | Pages 6-7 |
| Search strategy | 7 | Present the full search strategies for all databases, registers and websites, including any filters and limits used. | Additional Tables 2-4 |
| Selection process | 8 | Specify the methods used to decide whether a study met the inclusion criteria of the review, including how many reviewers screened each record and each report retrieved, whether they worked independently, and if applicable, details of automation tools used in the process. | Page 7 |
| Data collection process | 9 | Specify the methods used to collect data from reports, including how many reviewers collected data from each report, whether they worked independently, any processes for obtaining or confirming data from study investigators, and if applicable, details of automation tools used in the process. | Page 7 |
| Data items | 10a | List and define all outcomes for which data were sought. Specify whether all results that were compatible with each outcome domain in each study were sought (e.g. for all measures, time points, analyses), and if not, the methods used to decide which results to collect. | Table 1, Additional Table 9 |
|  | 10b | List and define all other variables for which data were sought (e.g. participant and intervention characteristics, funding sources). Describe any assumptions made about any missing or unclear information. | Table 2, Additional Tables 7-8 |
| Study risk of bias assessment | 11 | Specify the methods used to assess risk of bias in the included studies, including details of the tool(s) used, how many reviewers assessed each study and whether they worked independently, and if applicable, details of automation tools used in the process. | Page 7 |
| Effect measures | 12 | Specify for each outcome the effect measure(s) (e.g. risk ratio, mean difference) used in the synthesis or presentation of results. | Pages 7-8 |
| Synthesis methods | 13a | Describe the processes used to decide which studies were eligible for each synthesis (e.g. tabulating the study intervention characteristics and comparing against the planned groups for each synthesis (item #5)). | Pages 8-9, 11-12 |
|  | 13b | Describe any methods required to prepare the data for presentation or synthesis, such as handling of missing summary statistics, or data conversions. | Page 8 |
|  | 13c | Describe any methods used to tabulate or visually display results of individual studies and syntheses. | Page 8 |
|  | 13d | Describe any methods used to synthesize results and provide a rationale for the choice(s). If meta-analysis was performed, describe the model(s), method(s) to identify the presence and extent of statistical heterogeneity, and software package(s) used. | Pages 7-8 |
|  | 13e | Describe any methods used to explore possible causes of heterogeneity among study results (e.g. subgroup analysis, meta-regression). | Page 7 |
|  | 13f | Describe any sensitivity analyses conducted to assess robustness of the synthesized results. | NA |
| Reporting bias assessment | 14 | Describe any methods used to assess risk of bias due to missing results in a synthesis (arising from reporting biases). | NA |
| Certainty assessment | 15 | Describe any methods used to assess certainty (or confidence) in the body of evidence for an outcome. | NA |
| **RESULTS** | | |  |
| Study selection | 16a | Describe the results of the search and selection process, from the number of records identified in the search to the number of studies included in the review, ideally using a flow diagram. | Pages 8-9, Figure 1 |
|  | 16b | Cite studies that might appear to meet the inclusion criteria, but which were excluded, and explain why they were excluded. | Pages 8-9 |
| Study characteristics | 17 | Cite each included study and present its characteristics. | Table 2, Additional Table 2 |
| Risk of bias in studies | 18 | Present assessments of risk of bias for each included study. | NA |
| Results of individual studies | 19 | For all outcomes, present, for each study: (a) summary statistics for each group (where appropriate) and (b) an effect estimate and its precision (e.g. confidence/credible interval), ideally using structured tables or plots. | NA |
| Results of syntheses | 20a | For each synthesis, briefly summarise the characteristics and risk of bias among contributing studies. | Pages 11-12 |
|  | 20b | Present results of all statistical syntheses conducted. If meta-analysis was done, present for each the summary estimate and its precision (e.g. confidence/credible interval) and measures of statistical heterogeneity. If comparing groups, describe the direction of the effect. | Tables 3-5, Pages 14-16 |
|  | 20c | Present results of all investigations of possible causes of heterogeneity among study results. | Page 7, 11-12 |
|  | 20d | Present results of all sensitivity analyses conducted to assess the robustness of the synthesized results. | NA |
| Reporting biases | 21 | Present assessments of risk of bias due to missing results (arising from reporting biases) for each synthesis assessed. | NA |
| Certainty of evidence | 22 | Present assessments of certainty (or confidence) in the body of evidence for each outcome assessed. | NA |
| **DISCUSSION** | | |  |
| Discussion | 23a | Provide a general interpretation of the results in the context of other evidence. | Page 17 |
|  | 23b | Discuss any limitations of the evidence included in the review. | Pages 17-18 |
|  | 23c | Discuss any limitations of the review processes used. | Page 18 |
|  | 23d | Discuss implications of the results for practice, policy, and future research. | Page 18 |
| **OTHER INFORMATION** | | |  |
| Registration and protocol | 24a | Provide registration information for the review, including register name and registration number, or state that the review was not registered. | Page 5 |
|  | 24b | Indicate where the review protocol can be accessed, or state that a protocol was not prepared. | Page 19 |
|  | 24c | Describe and explain any amendments to information provided at registration or in the protocol. | NA |
| Support | 25 | Describe sources of financial or non-financial support for the review, and the role of the funders or sponsors in the review. | Page 19 |
| Competing interests | 26 | Declare any competing interests of review authors. | Page 19 |
| Availability of data, code and other materials | 27 | Report which of the following are publicly available and where they can be found: template data collection forms; data extracted from included studies; data used for all analyses; analytic code; any other materials used in the review. | Page 19 |

Additional Table 2. Search strategy for EMBASE 1974 to 2022 April 20

| No. | Criteria | Strings | Hits |
| --- | --- | --- | --- |
| 1 | Population | exp triple negative breast neoplasms/ | 28,744 |
| 2 |  | triple negative breast neoplasms.mp. | 289 |
| 3 |  | ("er-negative pr-negative her2-negative breast neoplasm*" or "er negative pr negative her2 negative breast neoplasm*" or "triple-negative breast neoplasm*" or "triple negative breast neoplasm*" or "triple-negative adj10 breast neoplasm*" or "triple negative adj10 breast neoplasm*" or "er-negative pr-negative her2-negative breast cancer*" or "er negative pr negative her2 negative breast cancer*" or "triple-negative breast cancer*" or "triple negative breast cancer*" or "triple-negative adj10 breast cancer*" or "triple negative adj10 breast cancer*" or "TNBC").mp. | 32,064 |
| 4 |  | or/1-3 | 32,064 |
| 5 |  | (breast and cancer*).mp. or breast neoplasm*.mp. or breast carcinoma*.mp. or ductal neoplasm*.mp. or ductal carcinoma*.mp. or lobular neoplasm*.mp. or lobular carcinoma*.mp. | 688,054 |
| 6 |  | 4 and 5 | 32,043 |
| 7 | Intervention | exp pembrolizumab/ | 26,207 |
| 8 |  | (pembrolizumab or lambrolizumab or keytruda or mk-3475 or mk3475 or mk 3475 or l01xc18).mp. | 27,588 |
| 9 |  | exp nivolumab/ | 28,090 |
| 10 |  | (nivolumab or opdivo or ono-4538 or ono 4538 or ono4538 or mdx-1106 or mdx 1106 or mdx1106 or bms-936558 or bms 936558 or bms936558).mp. | 29,403 |
| 11 |  | exp atezolizumab/ | 9,902 |
| 12 |  | (atezolizumab or anti-pdl1 or mpdl3280a or tecentriq or rg7446 or rg-7446).mp. | 11,131 |
| 13 |  | exp avelumab/ | 4,337 |
| 14 |  | (avelumab or msb0010718c).mp. | 4,522 |
| 15 |  | exp durvalumab/ | 6,849 |
| 16 |  | (durvalumab or medi4736 or medi-4736 or imfinzi).mp. | 7,147 |
| 17 |  | exp cemiplimab/ | 946 |
| 18 |  | (cemiplimab or regn2810).mp. | 1,010 |
| 19 |  | exp doxorubicin/ | 205,794 |
| 20 |  | (doxorubicin or farmiblastina or ribodoxo or rubex or adriamycin or adriblastin or adriblastine or adriblastina or adriablastine or adriablastin or adrimedac or doxo-cell or doxo cell or urokit doxo-cell or urokit doxo cell or doxolem or doxorubicin hexal or doxorubicin hydrochloride or hydrochloride, doxorubicin or doxorubicin nc or doxorubicina ferrer farm or doxorubicina funk or doxorubicina tedec or doxorubicine baxter or doxotec or myocet or onkodox).mp. | 217,446 |
| 21 |  | exp cyclophosphamide/ | 233,302 |
| 22 |  | (cyclophosphamide or cytophosphane or cyclophosphamide monohydrate or monohydrate, cyclophosphamide or cyclophosphane or cyclophosphamide anhydrous or anhydrous, cyclophosphamide or cytophosphan or endoxan or neosar or nsc-26271 or nsc 26271 or nsc26271 or procytox or sendoxan or b-518 or b 518 or b518 or cytoxan).mp. | 245,353 |
| 23 |  | exp docetaxel/ | 66,180 |
| 24 |  | (docetaxel or docetaxel hydrate or docetaxel trihydrate or docetaxol or docetaxel anhydrous or n-debenzoyl-n-tert-butoxycarbonyl-10-deacetyltaxol or taxoltere metro or taxotere or nsc 628503 or rp 56976 or rp-56976).mp. | 68,468 |
| 25 |  | exp cyclophosphamide/ and exp methotrexate/ and exp fluorouracil/ | 17,415 |
| 26 |  | ((cyclophosphamide or cytophosphane or cyclophosphamide monohydrate or monohydrate, cyclophosphamide or cyclophosphane or cyclophosphamide anhydrous or anhydrous, cyclophosphamide or cytophosphan or endoxan or neosar or nsc-26271 or nsc 26271 or nsc26271 or procytox or sendoxan or b-518 or b 518 or b518 or cytoxan) and (methotrexate or amethopterin or mexate or methotrexate sodium or sodium, methotrexate or methotrexate, sodium salt or methotrexate, disodium salt or methotrexate hydrate or hydrate, methotrexate or methotrexate, dicesium salt or dicesium salt methotrexate) and (fluorouracil or 5fu or 5-fu or 5-fluorouracil or 5 fluorouracil or fluoruracil or 5-fu lederle or 5 fu lederle or 5-fu medac or 5 fu medac or 5-hu hexal or 5 hu hexal or adrucil or carac or efudix or fluoro-uracile icn or fluoro uracile icn or efudex or fluoroplex or flurodex or fluorouracil mononitrate or fluorouracil monopotassium salt or fluorouracil monosodium salt or fluorouracil potassium salt or fluorouracil-gry or fluorouracil gry or fluorouracile dakota or dakota, fluorouracile or fluorouracilo ferrer far or fluracedyl or haemato-fu or haemato fu or neofluor or onkofluor or ribofluor or 5-fluorouracil-biosyn or 5 fluorouracil biosyn)).mp. | 17,687 |
| 27 |  | exp cyclophosphamide/ and exp doxorubicin/ and exp fluorouracil/ | 18,918 |
| 28 |  | ((cyclophosphamide or cytophosphane or cyclophosphamide monohydrate or monohydrate, cyclophosphamide or cyclophosphane or cyclophosphamide anhydrous or anhydrous, cyclophosphamide or cytophosphan or endoxan or neosar or nsc-26271 or nsc 26271 or nsc26271 or procytox or sendoxan or b-518 or b 518 or b518 or cytoxan) and (doxorubicin or farmiblastina or ribodoxo or rubex or adriamycin or adriblastin or adriblastine or adriblastina or adriablastine or adriablastin or adrimedac or doxo-cell or doxo cell or urokit doxo-cell or urokit doxo cell or doxolem or doxorubicin hexal or doxorubicin hydrochloride or hydrochloride, doxorubicin or doxorubicin nc or doxorubicina ferrer farm or doxorubicina funk or doxorubicina tedec or doxorubicine baxter or doxotec or myocet or onkodox) and (fluorouracil or 5fu or 5-fu or 5-fluorouracil or 5 fluorouracil or fluoruracil or 5-fu lederle or 5 fu lederle or 5-fu medac or 5 fu medac or 5-hu hexal or 5 hu hexal or adrucil or carac or efudix or fluoro-uracile icn or fluoro uracile icn or efudex or fluoroplex or flurodex or fluorouracil mononitrate or fluorouracil monopotassium salt or fluorouracil monosodium salt or fluorouracil potassium salt or fluorouracil-gry or fluorouracil gry or fluorouracile dakota or dakota, fluorouracile or fluorouracilo ferrer far or fluracedyl or haemato-fu or haemato fu or neofluor or onkofluor or ribofluor or 5-fluorouracil-biosyn or 5 fluorouracil biosyn)).mp. | 19,282 |
| 29 |  | exp fluorouracil/ and exp epirubicin/ and exp cyclophosphamide/ | 9,552 |
| 30 |  | ((fluorouracil or 5fu or 5-fu or 5-fluorouracil or 5 fluorouracil or fluoruracil or 5-fu lederle or 5 fu lederle or 5-fu medac or 5 fu medac or 5-hu hexal or 5 hu hexal or adrucil or carac or efudix or fluoro-uracile icn or fluoro uracile icn or efudex or fluoroplex or flurodex or fluorouracil mononitrate or fluorouracil monopotassium salt or fluorouracil monosodium salt or fluorouracil potassium salt or fluorouracil-gry or fluorouracil gry or fluorouracile dakota or dakota, fluorouracile or fluorouracilo ferrer far or fluracedyl or haemato-fu or haemato fu or neofluor or onkofluor or ribofluor or 5-fluorouracil-biosyn or 5 fluorouracil biosyn) and (epirubicin or epi-cell or epi cell or epicell or epilem or farmorubicina or imi-28 or imi 28 or imi28 or nsc-256942 or nsc 256942 or nsc256942 or ellence or pharmorubicin or farmorubicine or farmorubicin or epirubicin hydrochloride or hydrochloride, epirubicin) and (cyclophosphamide or cytophosphane or cyclophosphamide monohydrate or monohydrate, cyclophosphamide or cyclophosphane or cyclophosphamide anhydrous or anhydrous, cyclophosphamide or cytophosphan or endoxan or neosar or nsc-26271 or nsc 26271 or nsc26271 or procytox or sendoxan or b-518 or b 518 or b518 or cytoxan)).mp. | 9,758 |
| 31 |  | exp gemcitabine/ | 64,842 |
| 32 |  | (gemcitabine or dfdcyd or 2',2'-difluorodeoxycytidine or 2'-deoxy-2'-difluorocytidine or 2',2'-dfdc or 2',2'-difluoro-2'-deoxycytidine or gemcitabine hydrochloride or ly 188011 or ly-188011 or gemzar or 2'-deoxy-2',2''-difluorocytidine-5'-o-monophosphate).mp. | 67,259 |
| 33 |  | exp carboplatin/ | 78,197 |
| 34 |  | (carboplatin or cbdca or blastocarb or carbosin or carbotec or ercar or jm-8 or jm 8 or jm8 or neocarbo or nsc-241240 or nsc 241240 or nsc241240 or paraplatin or paraplatine or platinwas or ribocarbo or carboplat or nealorin).mp. | 81,065 |
| 35 |  | exp bevacizumab/ | 66,213 |
| 36 |  | (bevacizumab or avastin).mp. | 68,329 |
| 37 |  | exp paclitaxel/ | 120,393 |
| 38 |  | (paclitaxel or anzatax or nsc-125973 or nsc 125973 or nsc125973 or taxol or taxol a or bris taxol or taxol, bris or paxene or praxel or 7-epi-taxol or 7 epi taxol or onxol).mp. | 127,369 |
| 39 |  | exp nab-paclitaxel/ | 120,393 |
| 40 |  | (nab-paclitaxel or albumin-bound paclitaxel or albumin bound paclitaxel or paclitaxel, albumin-bound or protein-bound paclitaxel or paclitaxel, protein-bound or protein bound paclitaxel or abraxane or abi007 or abi-007 or "abi 007").mp. | 6,619 |
| 41 |  | exp capecitabine/ | 33,456 |
| 42 |  | (capecitabine or xeloda).mp. | 35,809 |
| 43 |  | exp vinorelbine/ | 4,131 |
| 44 |  | (vinorelbine or 5'-nor-anhydrovinblastine or navelbine or vinorelbine tartrate or kw 2307 or kw-2307).mp. | 19,900 |
| 45 |  | exp eribulin/ | 2,993 |
| 46 |  | (eribulin or e 7389 or e-7389 or er-086526 or er086526 or "er 086526" or er-86526 or halaven or nsc 707389 or nsc707389 or nsc-707389 or b 1793 or b-1793 or b 1939 or b-1939 or eribulin mesylate or eribulin monomethanesulfonate or eribulin mesilate).mp. | 3,158 |
| 47 |  | exp pegylated liposomal doxorubicin/ | 205,794 |
| 48 |  | (pegylated liposomal doxorubicin or pegylated liposomal doxorubicin or caelyx or lipodox or dox-sl or doxil).mp. | 5,101 |
| 49 |  | exp cisplatin/ | 201,996 |
| 50 |  | (cisplatin or platinum diamminodichloride or diamminodichloride, platinum or cis-platinum or cis platinum or dichlorodiammineplatinum or cis-diamminedichloroplatinum or cis diamminedichloroplatinum or nsc-119875 or platino or platinol or biocisplatinum or platidiam).mp. | 211,984 |
| 51 |  | exp epirubicin/ | 30,907 |
| 52 |  | (epirubicin or epi-cell or epi cell or epicell or epilem or farmorubicina or imi-28 or imi 28 or imi28 or nsc-256942 or nsc 256942 or nsc256942 or ellence or pharmorubicin or farmorubicine or farmorubicin or epirubicin hydrochloride or hydrochloride, epirubicin).mp. | 31,648 |
| 53 |  | exp ixabepilone/ | 1,855 |
| 54 |  | (ixabepilone or azaepothilone b or bms247550 or bms 247550 or bms-247550).mp. | 1,923 |
| 55 |  | exp sacituzumab/ or sacituzumab.mp. or trodelvy.mp. or immu-132 or hrs7-sn-38 or govitecan-hziy | 511 |
| 56 |  | or/7-55 | 757,568 |
| 57 | Study design | clinical trial/ | 1,034,003 |
| 58 |  | randomized controlled trial/ | 705,971 |
| 59 |  | controlled clinical trial/ | 465,519 |
| 60 |  | multicenter study/ | 321,845 |
| 61 |  | phase 3 clinical trial/ | 60,249 |
| 62 |  | phase 4 clinical trial/ | 4,734 |
| 63 |  | exp randomization/ | 94,052 |
| 64 |  | single blind procedure/ | 45,927 |
| 65 |  | double blind procedure/ | 194,276 |
| 66 |  | crossover procedure/ | 70,060 |
| 67 |  | placebo/ | 379,520 |
| 68 |  | randomi?ed controlled trial$.tw. | 283,525 |
| 69 |  | rct.tw. | 46,446 |
| 70 |  | study design (random$ adj2 allocat$).tw. | 49,725 |
| 71 |  | single blind$.tw. | 28,692 |
| 72 |  | double blind$.tw. | 229,454 |
| 73 |  | ((treble or triple) adj blind$).tw. | 1,544 |
| 74 |  | placebo$.tw. | 341,797 |
| 75 |  | prospective study/ | 761,051 |
| 76 |  | or/57-75 | 2,675,232 |
| 77 |  | case study/ | 84,976 |
| 78 |  | case report.tw. | 483,981 |
| 79 |  | abstract report/ or letter/ | 1,236,269 |
| 80 |  | conference proceeding.pt. | - |
| 81 |  | conference abstract.pt. | 4,374,598 |
| 82 |  | editorial.pt. | 724,047 |
| 83 |  | letter.pt. | 1,221,159 |
| 84 |  | note.pt. | 891,241 |
| 85 |  | or/77-84 | 7,718,691 |
| 86 |  | 76 not 85 | 1,945,899 |
| 87 | Combined criteria | 6 and 56 and 86 | 886 |
| 86 | Language | limit 87 to english | 873 |

Additional Table 3. Search strategy for Ovid MEDLINE(R) In-Process & Other Non-Indexed Citations, Ovid MEDLINE(R) Daily and Ovid MEDLINE(R) 1946 to April 20, 2021

| **No.** | **Criteria** | **Strings** | **Hits** |
| --- | --- | --- | --- |
| 1 | Population | exp triple negative breast neoplasms/ | 7,946 |
| 2 |  | triple negative breast neoplasms.mp. | 8,002 |
| 3 |  | ("er-negative pr-negative her2-negative breast neoplasm*" or "er negative pr negative her2 negative breast neoplasm*" or "triple-negative breast neoplasm*" or "triple negative breast neoplasm*" or "triple-negative adj10 breast neoplasm*" or "triple negative adj10 breast neoplasm*" or "er-negative pr-negative her2-negative breast cancer*" or "er negative pr negative her2 negative breast cancer*" or "triple-negative breast cancer*" or "triple negative breast cancer*" or "triple-negative adj10 breast cancer*" or "triple negative adj10 breast cancer*" or "TNBC").mp. | 14,857 |
| 4 |  | or/1-3 | 14,857 |
| 5 |  | (breast and cancer*).mp. or breast neoplasm*.mp. or breast carcinoma*.mp. or ductal neoplasm*.mp. or ductal carcinoma*.mp. or lobular neoplasm*.mp. or lobular carcinoma*.mp. | 448,631 |
| 6 |  | 4 and 5 | 14,829 |
| 7 | Intervention | (pembrolizumab or lambrolizumab or keytruda or mk-3475 or mk3475 or mk 3475 or l01xc18).mp. | 7,115 |
| 8 |  | exp nivolumab/ | 4,243 |
| 9 |  | (nivolumab or opdivo or ono-4538 or ono 4538 or ono4538 or mdx-1106 or mdx 1106 or mdx1106 or bms-936558 or bms 936558 or bms936558).mp. | 7,977 |
| 10 |  | (atezolizumab or anti-pdl1 or mpdl3280a or tecentriq or rg7446 or rg-7446).mp. | 2,360 |
| 11 |  | (avelumab or msb0010718c).mp. | 746 |
| 12 |  | (durvalumab or medi4736 or medi-4736 or imfinzi).mp. | 1,112 |
| 13 |  | (cemiplimab or regn2810).mp. | 237 |
| 14 |  | exp doxorubicin/ | 62,063 |
| 15 |  | (doxorubicin or farmiblastina or ribodoxo or rubex or adriamycin or adriblastin or adriblastine or adriblastina or adriablastine or adriablastin or adrimedac or doxo-cell or doxo cell or urokit doxo-cell or urokit doxo cell or doxolem or doxorubicin hexal or doxorubicin hydrochloride or hydrochloride, doxorubicin or doxorubicin nc or doxorubicina ferrer farm or doxorubicina funk or doxorubicina tedec or doxorubicine baxter or doxotec or myocet or onkodox).mp. | 81,044 |
| 16 |  | exp cyclophosphamide/ | 56,055 |
| 17 |  | (cyclophosphamide or cytophosphane or cyclophosphamide monohydrate or monohydrate, cyclophosphamide or cyclophosphane or cyclophosphamide anhydrous or anhydrous, cyclophosphamide or cytophosphan or endoxan or neosar or nsc-26271 or nsc 26271 or nsc26271 or procytox or sendoxan or b-518 or b 518 or b518 or cytoxan).mp. | 74,675 |
| 18 |  | exp docetaxel/ | 11,683 |
| 19 |  | (docetaxel or docetaxel hydrate or docetaxel trihydrate or docetaxol or docetaxel anhydrous or n-debenzoyl-n-tert-butoxycarbonyl-10-deacetyltaxol or taxoltere metro or taxotere or nsc 628503 or rp 56976 or rp-56976).mp. | 18,740 |
| 20 |  | exp cyclophosphamide/ and exp methotrexate/ and exp fluorouracil/ | 2,349 |
| 21 |  | ((cyclophosphamide or cytophosphane or cyclophosphamide monohydrate or monohydrate, cyclophosphamide or cyclophosphane or cyclophosphamide anhydrous or anhydrous, cyclophosphamide or cytophosphan or endoxan or neosar or nsc-26271 or nsc 26271 or nsc26271 or procytox or sendoxan or b-518 or b 518 or b518 or cytoxan) and (methotrexate or amethopterin or mexate or methotrexate sodium or sodium, methotrexate or methotrexate, sodium salt or methotrexate, disodium salt or methotrexate hydrate or hydrate, methotrexate or methotrexate, dicesium salt or dicesium salt methotrexate) and (fluorouracil or 5fu or 5-fu or 5-fluorouracil or 5 fluorouracil or fluoruracil or 5-fu lederle or 5 fu lederle or 5-fu medac or 5 fu medac or 5-hu hexal or 5 hu hexal or adrucil or carac or efudix or fluoro-uracile icn or fluoro uracile icn or efudex or fluoroplex or flurodex or fluorouracil mononitrate or fluorouracil monopotassium salt or fluorouracil monosodium salt or fluorouracil potassium salt or fluorouracil-gry or fluorouracil gry or fluorouracile dakota or dakota, fluorouracile or fluorouracilo ferrer far or fluracedyl or haemato-fu or haemato fu or neofluor or onkofluor or ribofluor or 5-fluorouracil-biosyn or 5 fluorouracil biosyn)).mp. | 3,201 |
| 22 |  | exp cyclophosphamide/ and exp doxorubicin/ and exp fluorouracil/ | 2,559 |
| 23 |  | ((cyclophosphamide or cytophosphane or cyclophosphamide monohydrate or monohydrate, cyclophosphamide or cyclophosphane or cyclophosphamide anhydrous or anhydrous, cyclophosphamide or cytophosphan or endoxan or neosar or nsc-26271 or nsc 26271 or nsc26271 or procytox or sendoxan or b-518 or b 518 or b518 or cytoxan) and (doxorubicin or farmiblastina or ribodoxo or rubex or adriamycin or adriblastin or adriblastine or adriblastina or adriablastine or adriablastin or adrimedac or doxo-cell or doxo cell or urokit doxo-cell or urokit doxo cell or doxolem or doxorubicin hexal or doxorubicin hydrochloride or hydrochloride, doxorubicin or doxorubicin nc or doxorubicina ferrer farm or doxorubicina funk or doxorubicina tedec or doxorubicine baxter or doxotec or myocet or onkodox) and (fluorouracil or 5fu or 5-fu or 5-fluorouracil or 5 fluorouracil or fluoruracil or 5-fu lederle or 5 fu lederle or 5-fu medac or 5 fu medac or 5-hu hexal or 5 hu hexal or adrucil or carac or efudix or fluoro-uracile icn or fluoro uracile icn or efudex or fluoroplex or flurodex or fluorouracil mononitrate or fluorouracil monopotassium salt or fluorouracil monosodium salt or fluorouracil potassium salt or fluorouracil-gry or fluorouracil gry or fluorouracile dakota or dakota, fluorouracile or fluorouracilo ferrer far or fluracedyl or haemato-fu or haemato fu or neofluor or onkofluor or ribofluor or 5-fluorouracil-biosyn or 5 fluorouracil biosyn)).mp. | 2,631 |
| 24 |  | exp fluorouracil/ and exp epirubicin/ and exp cyclophosphamide/ | 936 |
| 25 |  | ((fluorouracil or 5fu or 5-fu or 5-fluorouracil or 5 fluorouracil or fluoruracil or 5-fu lederle or 5 fu lederle or 5-fu medac or 5 fu medac or 5-hu hexal or 5 hu hexal or adrucil or carac or efudix or fluoro-uracile icn or fluoro uracile icn or efudex or fluoroplex or flurodex or fluorouracil mononitrate or fluorouracil monopotassium salt or fluorouracil monosodium salt or fluorouracil potassium salt or fluorouracil-gry or fluorouracil gry or fluorouracile dakota or dakota, fluorouracile or fluorouracilo ferrer far or fluracedyl or haemato-fu or haemato fu or neofluor or onkofluor or ribofluor or 5-fluorouracil-biosyn or 5 fluorouracil biosyn) and (epirubicin or epi-cell or epi cell or epicell or epilem or farmorubicina or imi-28 or imi 28 or imi28 or nsc-256942 or nsc 256942 or nsc256942 or ellence or pharmorubicin or farmorubicine or farmorubicin or epirubicin hydrochloride or hydrochloride, epirubicin) and (cyclophosphamide or cytophosphane or cyclophosphamide monohydrate or monohydrate, cyclophosphamide or cyclophosphane or cyclophosphamide anhydrous or anhydrous, cyclophosphamide or cytophosphan or endoxan or neosar or nsc-26271 or nsc 26271 or nsc26271 or procytox or sendoxan or b-518 or b 518 or b518 or cytoxan)).mp. | 1,396 |
| 26 |  | (gemcitabine or dfdcyd or 2',2'-difluorodeoxycytidine or 2'-deoxy-2'-difluorocytidine or 2',2'-dfdc or 2',2'-difluoro-2'-deoxycytidine or gemcitabine hydrochloride or ly 188011 or ly-188011 or gemzar or 2'-deoxy-2',2''-difluorocytidine-5'-o-monophosphate).mp. | 19,304 |
| 27 |  | exp carboplatin/ | 12,550 |
| 28 |  | (carboplatin or cbdca or blastocarb or carbosin or carbotec or ercar or jm-8 or jm 8 or jm8 or neocarbo or nsc-241240 or nsc 241240 or nsc241240 or paraplatin or paraplatine or platinwas or ribocarbo or carboplat or nealorin).mp. | 19,519 |
| 29 |  | exp bevacizumab/ | 13,224 |
| 30 |  | (bevacizumab or avastin).mp. | 21,048 |
| 31 |  | exp paclitaxel/ | 29,440 |
| 32 |  | (paclitaxel or anzatax or nsc-125973 or nsc 125973 or nsc125973 or taxol or taxol a or bris taxol or taxol, bris or paxene or praxel or 7-epi-taxol or 7 epi taxol or onxol).mp. | 44,086 |
| 33 |  | (nab-paclitaxel or albumin-bound paclitaxel or albumin bound paclitaxel or paclitaxel, albumin-bound or protein-bound paclitaxel or paclitaxel, protein-bound or protein bound paclitaxel or abraxane or abi007 or abi-007 or "abi 007").mp. | 2,465 |
| 34 |  | exp capecitabine/ | 5,111 |
| 35 |  | (capecitabine or xeloda).mp. | 8,195 |
| 36 |  | exp vinorelbine/ | 2,810 |
| 37 |  | (vinorelbine or 5'-nor-anhydrovinblastine or navelbine or vinorelbine tartrate or kw 2307 or kw-2307).mp. | 4,362 |
| 38 |  | (eribulin or e 7389 or e-7389 or er-086526 or er086526 or "er 086526" or er-86526 or halaven or nsc 707389 or nsc707389 or nsc-707389 or b 1793 or b-1793 or b 1939 or b-1939 or eribulin mesylate or eribulin monomethanesulfonate or eribulin mesilate).mp. | 872 |
| 39 |  | (pegylated liposomal doxorubicin or pegylated liposomal doxorubicin or caelyx or lipodox or dox-sl or doxil).mp. | 1,838 |
| 40 |  | exp cisplatin/ | 56,161 |
| 41 |  | (cisplatin or platinum diamminodichloride or diamminodichloride, platinum or cis-platinum or cis platinum or dichlorodiammineplatinum or cis-diamminedichloroplatinum or cis diamminedichloroplatinum or nsc-119875 or platino or platinol or biocisplatinum or platidiam).mp. | 83,826 |
| 42 |  | exp epirubicin/ | 5,396 |
| 43 |  | (epirubicin or epi-cell or epi cell or epicell or epilem or farmorubicina or imi-28 or imi 28 or imi28 or nsc-256942 or nsc 256942 or nsc256942 or ellence or pharmorubicin or farmorubicine or farmorubicin or epirubicin hydrochloride or hydrochloride, epirubicin).mp. | 7,719 |
| 44 |  | (ixabepilone or azaepothilone b or bms247550 or bms 247550 or bms-247550).mp. | 458 |
| 45 |  | (sacituzumab or trodelvy or immu-132 or hrs7-sn-38 or govitecan-hziy).ti,ab. | 140 |
| 46 |  | or/7-45 | 308,317 |
| 47 | Study design | randomized controlled trials as topic/ | 154,599 |
| 48 |  | randomized controlled trial/ | 565,606 |
| 49 |  | random allocation/ | 106,834 |
| 50 |  | double blind method/ | 171,321 |
| 51 |  | single blind method/ | 31,841 |
| 52 |  | clinical trial/ | 534,766 |
| 53 |  | clinical trial, phase i.pt. | 23,734 |
| 54 |  | clinical trial, phase II.pt. | 37,810 |
| 55 |  | clinical trial, phase III.pt. | 20,417 |
| 56 |  | clinical trial, phase iv.pt. | 2,316 |
| 57 |  | controlled clinical trial.pt. | 94,827 |
| 58 |  | randomized controlled trial.pt. | 565,606 |
| 59 |  | multicenter study.pt. | 319,870 |
| 60 |  | clinical trial.pt. | 534,766 |
| 61 |  | exp clinical trials as topic/ | 373,005 |
| 62 |  | or/47-61 | 1,509,082 |
| 63 |  | (clinical adj trial$).tw. | 432,774 |
| 64 |  | ((singl$ or doubl$ or treb$ or tripl$) adj (blind$3 or mask$3)).tw. | 187,712 |
| 65 |  | placebos/ | 35,919 |
| 66 |  | placebo$.tw. | 234,718 |
| 67 |  | randomly allocated.tw. | 33,301 |
| 68 |  | (allocated adj2 random$).tw. | 36,880 |
| 69 |  | or/63-68 | 723,234 |
| 70 |  | 62 or 69 | 1,818,119 |
| 71 |  | case report.tw. | 360,091 |
| 72 |  | letter/ | 1,176,989 |
| 73 |  | historical article/ | 368,252 |
| 74 |  | or/71-73 | 1,887,628 |
| 75 |  | 70 not 74 | 1,777,099 |
| 76 | Combined criteria | 6 and 46 and 75 | 613 |
| 77 | Language | limit 76 to english | 601 |

Additional Table 4. Search strategy for EBM Reviews - Cochrane Central Register of Controlled Trials March 2022

| **No.** | **Criteria** | **Strings** | **Hits** |
| --- | --- | --- | --- |
| 1 | Population | exp triple negative breast neoplasms/ | 351 |
| 2 |  | triple negative breast neoplasms.mp. | 357 |
| 3 |  | ("er-negative pr-negative her2-negative breast neoplasm*" or "er negative pr negative her2 negative breast neoplasm*" or "triple-negative breast neoplasm*" or "triple negative breast neoplasm*" or "triple-negative adj10 breast neoplasm*" or "triple negative adj10 breast neoplasm*" or "er-negative pr-negative her2-negative breast cancer*" or "er negative pr negative her2 negative breast cancer*" or "triple-negative breast cancer*" or "triple negative breast cancer*" or "triple-negative adj10 breast cancer*" or "triple negative adj10 breast cancer*" or "TNBC").mp. | 1,574 |
| 4 |  | or/1-3 | 1,574 |
| 5 |  | (breast and cancer*).mp. or breast neoplasm*.mp. or breast carcinoma*.mp. or ductal neoplasm*.mp. or ductal carcinoma*.mp. or lobular neoplasm*.mp. or lobular carcinoma*.mp. | 43,413 |
| 6 |  | 4 and 5 | 1,572 |
| 7 | Intervention | (pembrolizumab or lambrolizumab or keytruda or mk-3475 or mk3475 or mk 3475 or l01xc18).mp. | 2,339 |
| 8 |  | (nivolumab or opdivo or ono-4538 or ono 4538 or ono4538 or mdx-1106 or mdx 1106 or mdx1106 or bms-936558 or bms 936558 or bms936558).mp. | 2,417 |
| 9 |  | (atezolizumab or anti-pdl1 or mpdl3280a or tecentriq or rg7446 or rg-7446).mp. | 1,173 |
| 10 |  | (avelumab or msb0010718c).mp. | 309 |
| 11 |  | (durvalumab or medi4736 or medi-4736 or imfinzi).mp. | 846 |
| 12 |  | (cemiplimab or regn2810).mp. | 76 |
| 13 |  | exp doxorubicin/ | 4,989 |
| 14 |  | (doxorubicin or farmiblastina or ribodoxo or rubex or adriamycin or adriblastin or adriblastine or adriblastina or adriablastine or adriablastin or adrimedac or doxo-cell or doxo cell or urokit doxo-cell or urokit doxo cell or doxolem or doxorubicin hexal or doxorubicin hydrochloride or hydrochloride, doxorubicin or doxorubicin nc or doxorubicina ferrer farm or doxorubicina funk or doxorubicina tedec or doxorubicine baxter or doxotec or myocet or onkodox).mp. | 9,318 |
| 15 |  | exp cyclophosphamide/ | 5,662 |
| 16 |  | (cyclophosphamide or cytophosphane or cyclophosphamide monohydrate or monohydrate, cyclophosphamide or cyclophosphane or cyclophosphamide anhydrous or anhydrous, cyclophosphamide or cytophosphan or endoxan or neosar or nsc-26271 or nsc 26271 or nsc26271 or procytox or sendoxan or b-518 or b 518 or b518 or cytoxan).mp. | 13,260 |
| 17 |  | (docetaxel or docetaxel hydrate or docetaxel trihydrate or docetaxol or docetaxel anhydrous or n-debenzoyl-n-tert-butoxycarbonyl-10-deacetyltaxol or taxoltere metro or taxotere or nsc 628503 or rp 56976 or rp-56976).mp. | 8,105 |
| 18 |  | exp cyclophosphamide/ and exp methotrexate/ and exp fluorouracil/ | 628 |
| 19 |  | ((cyclophosphamide or cytophosphane or cyclophosphamide monohydrate or monohydrate, cyclophosphamide or cyclophosphane or cyclophosphamide anhydrous or anhydrous, cyclophosphamide or cytophosphan or endoxan or neosar or nsc-26271 or nsc 26271 or nsc26271 or procytox or sendoxan or b-518 or b 518 or b518 or cytoxan) and (methotrexate or amethopterin or mexate or methotrexate sodium or sodium, methotrexate or methotrexate, sodium salt or methotrexate, disodium salt or methotrexate hydrate or hydrate, methotrexate or methotrexate, dicesium salt or dicesium salt methotrexate) and (fluorouracil or 5fu or 5-fu or 5-fluorouracil or 5 fluorouracil or fluoruracil or 5-fu lederle or 5 fu lederle or 5-fu medac or 5 fu medac or 5-hu hexal or 5 hu hexal or adrucil or carac or efudix or fluoro-uracile icn or fluoro uracile icn or efudex or fluoroplex or flurodex or fluorouracil mononitrate or fluorouracil monopotassium salt or fluorouracil monosodium salt or fluorouracil potassium salt or fluorouracil-gry or fluorouracil gry or fluorouracile dakota or dakota, fluorouracile or fluorouracilo ferrer far or fluracedyl or haemato-fu or haemato fu or neofluor or onkofluor or ribofluor or 5-fluorouracil-biosyn or 5 fluorouracil biosyn)).mp. | 1,200 |
| 20 |  | exp cyclophosphamide/ and exp doxorubicin/ and exp fluorouracil/ | 770 |
| 21 |  | ((cyclophosphamide or cytophosphane or cyclophosphamide monohydrate or monohydrate, cyclophosphamide or cyclophosphane or cyclophosphamide anhydrous or anhydrous, cyclophosphamide or cytophosphan or endoxan or neosar or nsc-26271 or nsc 26271 or nsc26271 or procytox or sendoxan or b-518 or b 518 or b518 or cytoxan) and (doxorubicin or farmiblastina or ribodoxo or rubex or adriamycin or adriblastin or adriblastine or adriblastina or adriablastine or adriablastin or adrimedac or doxo-cell or doxo cell or urokit doxo-cell or urokit doxo cell or doxolem or doxorubicin hexal or doxorubicin hydrochloride or hydrochloride, doxorubicin or doxorubicin nc or doxorubicina ferrer farm or doxorubicina funk or doxorubicina tedec or doxorubicine baxter or doxotec or myocet or onkodox) and (fluorouracil or 5fu or 5-fu or 5-fluorouracil or 5 fluorouracil or fluoruracil or 5-fu lederle or 5 fu lederle or 5-fu medac or 5 fu medac or 5-hu hexal or 5 hu hexal or adrucil or carac or efudix or fluoro-uracile icn or fluoro uracile icn or efudex or fluoroplex or flurodex or fluorouracil mononitrate or fluorouracil monopotassium salt or fluorouracil monosodium salt or fluorouracil potassium salt or fluorouracil-gry or fluorouracil gry or fluorouracile dakota or dakota, fluorouracile or fluorouracilo ferrer far or fluracedyl or haemato-fu or haemato fu or neofluor or onkofluor or ribofluor or 5-fluorouracil-biosyn or 5 fluorouracil biosyn)).mp. | 970 |
| 22 |  | exp fluorouracil/ and exp epirubicin/ and exp cyclophosphamide/ | 376 |
| 23 |  | ((fluorouracil or 5fu or 5-fu or 5-fluorouracil or 5 fluorouracil or fluoruracil or 5-fu lederle or 5 fu lederle or 5-fu medac or 5 fu medac or 5-hu hexal or 5 hu hexal or adrucil or carac or efudix or fluoro-uracile icn or fluoro uracile icn or efudex or fluoroplex or flurodex or fluorouracil mononitrate or fluorouracil monopotassium salt or fluorouracil monosodium salt or fluorouracil potassium salt or fluorouracil-gry or fluorouracil gry or fluorouracile dakota or dakota, fluorouracile or fluorouracilo ferrer far or fluracedyl or haemato-fu or haemato fu or neofluor or onkofluor or ribofluor or 5-fluorouracil-biosyn or 5 fluorouracil biosyn) and (epirubicin or epi-cell or epi cell or epicell or epilem or farmorubicina or imi-28 or imi 28 or imi28 or nsc-256942 or nsc 256942 or nsc256942 or ellence or pharmorubicin or farmorubicine or farmorubicin or epirubicin hydrochloride or hydrochloride, epirubicin) and (cyclophosphamide or cytophosphane or cyclophosphamide monohydrate or monohydrate, cyclophosphamide or cyclophosphane or cyclophosphamide anhydrous or anhydrous, cyclophosphamide or cytophosphan or endoxan or neosar or nsc-26271 or nsc 26271 or nsc26271 or procytox or sendoxan or b-518 or b 518 or b518 or cytoxan)).mp. | 999 |
| 24 |  | (gemcitabine or dfdcyd or 2',2'-difluorodeoxycytidine or 2'-deoxy-2'-difluorocytidine or 2',2'-dfdc or 2',2'-difluoro-2'-deoxycytidine or gemcitabine hydrochloride or ly 188011 or ly-188011 or gemzar or 2'-deoxy-2',2''-difluorocytidine-5'-o-monophosphate).mp. | 6,632 |
| 25 |  | exp carboplatin/ | 2,567 |
| 26 |  | (carboplatin or cbdca or blastocarb or carbosin or carbotec or ercar or jm-8 or jm 8 or jm8 or neocarbo or nsc-241240 or nsc 241240 or nsc241240 or paraplatin or paraplatine or platinwas or ribocarbo or carboplat or nealorin).mp. | 8,026 |
| 27 |  | (bevacizumab or avastin).mp. | 7,189 |
| 28 |  | exp paclitaxel/ | 3,799 |
| 29 |  | (paclitaxel or anzatax or nsc-125973 or nsc 125973 or nsc125973 or taxol or taxol a or bris taxol or taxol, bris or paxene or praxel or 7-epi-taxol or 7 epi taxol or onxol).mp. | 11,669 |
| 30 |  | (nab-paclitaxel or albumin-bound paclitaxel or albumin bound paclitaxel or paclitaxel, albumin-bound or protein-bound paclitaxel or paclitaxel, protein-bound or protein bound paclitaxel or abraxane or abi007 or abi-007 or "abi 007").mp. | 2,682 |
| 31 |  | (capecitabine or xeloda).mp. | 4,462 |
| 32 |  | (vinorelbine or 5'-nor-anhydrovinblastine or navelbine or vinorelbine tartrate or kw 2307 or kw-2307).mp. | 1,969 |
| 33 |  | (eribulin or e 7389 or e-7389 or er-086526 or er086526 or "er 086526" or er-86526 or halaven or nsc 707389 or nsc707389 or nsc-707389 or b 1793 or b-1793 or b 1939 or b-1939 or eribulin mesylate or eribulin monomethanesulfonate or eribulin mesilate).mp. | 427 |
| 34 |  | (pegylated liposomal doxorubicin or pegylated liposomal doxorubicin or caelyx or lipodox or dox-sl or doxil).mp. | 630 |
| 35 |  | exp cisplatin/ | 5,237 |
| 36 |  | (cisplatin or platinum diamminodichloride or diamminodichloride, platinum or cis-platinum or cis platinum or dichlorodiammineplatinum or cis-diamminedichloroplatinum or cis diamminedichloroplatinum or nsc-119875 or platino or platinol or biocisplatinum or platidiam).mp. | 15,643 |
| 37 |  | exp epirubicin/ | 1,245 |
| 38 |  | (epirubicin or epi-cell or epi cell or epicell or epilem or farmorubicina or imi-28 or imi 28 or imi28 or nsc-256942 or nsc 256942 or nsc256942 or ellence or pharmorubicin or farmorubicine or farmorubicin or epirubicin hydrochloride or hydrochloride, epirubicin).mp. | 3,392 |
| 39 |  | (ixabepilone or azaepothilone b or bms247550 or bms 247550 or bms-247550).mp. | 144 |
| 40 |  | (sacituzumab or trodelvy or immu-132 or hrs7-sn-38 or govitecan-hziy).mp. | 51 |
| 41 |  | or/7-40 | 60,125 |
| 42 | Combined criteria | 6 and 41 | 1,168 |
| 43 | Language | limit 42 to english language | 1,080 |

Additional Table 5. Trials identified in the systematic review

| **Trial ID** | **Registry number** | **Primary publication** | **Associated publications** |
| --- | --- | --- | --- |
| ETNA | NCT01822314 | Gianni et al. 2018^1^ | -- |
| GeparSepto | NCT01583426 | Untch et al. 2016^2^ | Untch et al. 2019^3^ |
| IMpassion031 | NCT03197935 | Mittendorf et al. 2020^4^ | Mittendorf et al. 2021^5^ |
| KEYNOTE-522 | NCT03036488 | Schmid et al. 2022^6^ | Schmid et al. 2020^7^ |
|  |  |  | Clinical study report* |
|  |  |  | Dent et al. 2020^8^ |
| NATT | -- | Chen et al. 2013^9^ | Chen et al. 2016^10^ |
| NCI 10013 | NCT02883062 | Ademuyiwa et al.^11^ | -- |
| Vriens 2013 | NCT00314977 | Vriens et al. 2013^12^ | -- |

*Provided by Merck & Co., Inc.

Additional Table 6. Trials included in the feasibility assessment and network meta-analysis

| **Trial ID** | **Registry number** | **Primary publication** | **Associated publications** |
| --- | --- | --- | --- |
| KEYNOTE-522 | NCT03036488 | Clinical study report* | -- |
| Alliance (CALGB 40603)** | NCT00861705 | Clinicaltrials.gov^13^ | Sikov et al. 2015^14^ |
| GeparSepto | NCT01583426 | Untch et al. 2016^2^ | Untch et al. 2019^3^ |
| neoSTOP** | NCT02413320 | Sharma et al. 2021^15^ | -- |
| BrighTNess** | NCT02032277 | Loibl et al. 2021^16^ | Loibl et al. 2018^17^ |

*Provided by Merck & Co, Inc. **Trials identified by the targeted literature search.

Additional Table 7. Treatment characteristics of included trials

| **Trial ID** | **Treatment** | **Treatment phase 1** | **Treatment phase 2** | **Treatment phase 3** |
| --- | --- | --- | --- | --- |
| **BrighTNess** | Pac+carbo+veli followed by dox+cyclo | Pac IV 80 mg/m2; D1; 1-week cycle for 12 cycles for up to 16 weeks + carbo IV 6 AUC; D1; 3-week cycle for 4 cycles + veli PO 50 mg BID | Dox IV 60 mg/m2 + cyclo IV 600mg/m2; D1; 2- or 3-week cycle for 4 cycles | -- |
|  | Pac+carb followed by dox+cyclo | Pac IV 80 mg/m2; D1; 1-week cycle for 12 cycles for up to 16 weeks + placebo IV 6 AUC; D1; 3-week cycle for 4 cycles + veli PO 50 mg BID | Dox IV 60 mg/m2 + cyclo IV 600mg/m2; D1; 2- or 3-week cycle for 4 cycles | -- |
|  | Pac followed by dox+cyclo | Pac IV 80 mg/m2; D1; 1-week cycle for 12 cycles for up to 16 weeks + placebo IV 6 AUC; D1; 3-week cycle for 4 cycles + placebo PO 50 mg BID | Dox IV 60 mg/m2 + cyclo IV 600mg/m2; D1; 2- or 3-week cycle for 4 cycles | -- |
| **Alliance (CALGB 40603)** | Pac followed by dox+cyclo | Pac IV 80 mg/m2; D1; 1-week cycle for 12 cycles | Dox IV 60 mg/m2 + cyclo IV 600mg/m2; D1; 2-week cycle for 4 cycles | -- |
|  | Pac+bev followed by dox+cyclo | Pac IV 80 mg/m2; D1; 1-week cycle for 12 cycles | Dox IV 60 mg/m2 + cyclo IV 600mg/m2; D1; 2-week cycle for 4 cycles | Bev IV 10 mg/kg; D1; 2-week cycle for 9 cycles |
|  | Pac+carb followed by dox+cyclo | Pac IV 80 mg/m2; D1; 1-week cycle for 12 cycles | Dox IV 60 mg/m2 + cyclo IV 600mg/m2; D1; 2-week cycle for 4 cycles following pac | Carb IV 6 AUC; D1; 3-week cycle for 4 cycles |
|  | Pac+bev+carb followed by dox+cyclo | Pac IV 80 mg/m2; D1; 1-week cycle for 12 cycles | Dox IV 60 mg/m2 + cyclo IV 600mg/m2; D1; 2-week cycle for 4 cycles | Carb IV 6 AUC; D1; 3-week cycle for 4 cycles + bev IV 10 mg/kg; D1; 2-week cycle for 9 cycles |
| **GeparSepto** | Nab-pac followed by epi+cyclo | Nab-pac IV 125 mg/m^2^; D1; 1-week cycle for 12 cycles | Epi IV 90mg/m^2^ + cyclo IV 600mg/m^2^; D1; 3-week cycle for 4 cycles | -- |
|  | Pac followed by epi + cyclo | Pac IV 80 mg/m^2^; D1; 1-week cycles for 12 cycles | Epi IV 90mg/m^2^ + cyclo IV 600mg/m^2^; D1; 3-week cycle for 4 cycles | -- |
| **KEYNOTE-522** | Pembro+pac+carb followed by pembro+dox OR epi+cyclo followed by adjuvant pembro | Pembro IV 200 mg/m^2^ + pac IV 80mg/m2 + carb IV AUC 5 or AUC 1.5; D1; 21-day cycle for 4 cycles | Pembro IV 200 mg/m^2^ + dox IV 60 mg/m^2^ or epi IV 90 mg/m2 + cyclo 600mg/m^2^; D1; 21-day cycle for 4 cycles | Post-surgery: Pembro IV 200mg/m^2^; D1; 21-day cycle for 9 cycles |
|  | Pac+carb followed by dox OR epi+cyclo | Placebo IV + pac IV 80mg/m^2^ + carb IV AUC 5 or AUC 1.5; D1; 21-day cycle for 4 cycles | Placebo + dox IV 60 mg/m2 or epi IV 90 mg/m^2^ + cyclo 600mg/m2; D1; 21-day cycle for 4 cycles | Post-surgery: Placebo; D1; 21-day cycle for 9 cycles |
| **NeoSTOP** | Pac+carb followed by dox+cyclo | Pac IV 80 mg/m2; D1; 1-week cycle for 12 cycles + carb IV 6 AUC; D1; 3-week cycle for 4 cycles | Dox IV 60 mg/m2 + cyclo IV 600mg/m2; D1; 2-week cycle for 4 cycles | -- |
|  | Doc+carbo followed by dox+cyclo | Doc IV 75 mg/m2 + carb IV 6 AUC; D1; 3-week cycle for 6 cycles | Dox IV 60 mg/m2 + cyclo IV 600mg/m2; D1; 2-week cycle for 4 cycles | -- |

**Abbreviations:** AUC = area under curve; bev = bevacizumab; BID = twice a day; carb = carboplatin; cyclo = cyclophosphamide; D = day; doc = docetaxel; IV = intravenous; mg/m^2^ = milligram per square meter; nab-pac = nab-paclitaxel; pac = paclitaxel; pembro = pembrolizumab; PO = oral; veli = veliparib.

Supplementary Table 8. Baseline patient characteristics of included trials

| **Trial ID** | **Treatment** | **N** | **Median age (range)** | **Female (%)** | **Caucasian (%)** | **ECOG PS 0 (%)** | **ECOG PS 1 (%)** |
| --- | --- | --- | --- | --- | --- | --- | --- |
| **BrighTNess** | Pac+carb+veli🡪anthra+cyclo | 316 | 51 (41-59) | **--** | **--** | **--** | **--** |
|  | Pac+carb🡪anthra+cyclo | 160 | 49 (40-57) | **--** | **--** | **--** | **--** |
|  | Pac🡪anthra+cyclo | 158 | 50 (42-59) | **--** | **--** | **--** | **--** |
| **Alliance (CALGB 40603)** | Pac🡪anthra+cyclo | 108 | -- | -- | 79 (73) | -- | -- |
|  | Pac+bev🡪anthra+cyclo+bev | 110 | -- | -- | 81 (74) | -- | -- |
|  | Pac+carb🡪anthra+cyclo | 113 | -- | -- | 80 (71) | -- | -- |
|  | Pac+carb+bev🡪anthra+cyclo+bev | 112 | -- | -- | 80 (71) | -- | -- |
| **GeparSepto** | Nab-pac🡪anthra+cyclo | 606 | 49 (--) | 606 (100) | -- | -- | -- |
|  | Pac🡪anthra+cyclo | 600 | 50 (--) | 600 (100) | -- | -- | -- |
| **KEYNOTE-522** | Pembro+pac+carb🡪  pembro+anthra+cyclo🡪adjuvant pembro | 784 | 49 (22-80) | 783 (99.9) | -- | -- | -- |
|  | Pac+carb-->anthra+cyclo | 390 | 48 (24-79) | 390 (100) | -- | -- | -- |
| **NeoSTOP** | Pac+carb-->anthra+cyclo | 48 | 51 (32-69) | -- | 35 (73) | -- | -- |
|  | Doc+carb-->anthra+cyclo | 52 | 54 (29-70) | -- | 36 (69) | -- | -- |

**Abbreviations:** Anthra = anthracycline; Bev = bevacizumab; Carb = carboplatin; Cyclo = cyclophosphamide; Doc = docetaxel; ECOG = Eastern Cooperative Oncology Group; ITT = intention-to-treat; N = number; Nab-pac = nab-paclitaxel; Pac = paclitaxel; Pembro = pembrolizumab; PS = performance status; Veli = veliparib.

**Notes:** Dashes (--) indicate where data were not reported. Arrows (**-->**) indicate where treatment was administered sequentially. Treatments to the left of the arrow were administered first. Anthracycline includes doxorubicin and epirubicin, which were assumed to be equivalent.

Additional Table 9. Definitions of pathological complete response, overall survival, and event-free survival used in the included trials

| **Trial ID** | **pCR** | **OS** | **EFS*** |
| --- | --- | --- | --- |
| **KEYNOTE-522** | Percentage of participants without residual invasive cancer on hematoxylin and eosin evaluation of the complete resected breast specimen and all sampled regional lymph nodes following completion of neoadjuvant systemic therapy by current AJCC staging criteria assessed by the local pathologist at the time of definitive surgery. | Time from randomization to death. | Time from randomization to any of the following events: progression of disease that precludes surgery, local or distant recurrence, second primary malignancy (breast or other cancers) or death. |
| **Alliance (CALGB 40603)** | Absence of residual invasive carcinoma in the breast (ypT0/is). | Number of patients who died due to any cause. | Time from definitive surgery to first instance of ipsilateral invasive breast tumor recurrence, local/regional invasive breast cancer recurrence, distant recurrence, or death from any cause. |
| **GeparSepto** | No microscopic evidence of residual invasive or non-invasive viable tumor cells in all resected specimens of the breast and axilla. | Time from registration and first event and will be analyzed after the end of the study by referring to data from the German Breast Group patient registry. | Disease progression during neoadjuvant therapy resulting in inoperability, any invasive locoregional (ipsilateral breast, locoregional lymph nodes) recurrence of disease after neoadjuvant therapy, any invasive contralateral breast cancer, any distant recurrence of disease, or death. |
| **NeoSTOP** | Absence of residual invasive disease in the breast and axilla, with or without ductal carcinoma in situ (ypT0/isN0). | Time from diagnosis to death as a result of any cause. | Time from diagnosis to first recurrence (invasive ipsilateral breast, invasive local/regional, or distant) or to breast cancer-related death. |
| **BrighTNess** | Absence of residual invasive disease on  evaluation of the resected breast specimen and resected lymph nodes following completion of neoadjuvant systemic therapy (i.e., ypT0/is ypN0 per the American Joint Committee on Cancer staging system). | Number of days from the day of randomisation to the date of death. | Time from randomisation to documentation of the first of the following events: failure to reach potential curative surgery; local, regional, or distant invasive recurrence of breast cancer following curative surgery; a new breast cancer or secondary malignancy; or death from any cause. |

**Abbreviations:** AJCC = American Joint Committee on Cancer; EFS = event-free survival; OS = overall survival; pCR; pathologic complete response.

*Reported as recurrence-free survival in Alliance (CALGB 40603).

Additional Table 10. Data sources for feasibility assessment and network meta-analysis

| **Trial ID** | **pCR (%)** | **EFS (HR)** | **EFS (KM)** | **OS (HR)** | **OS (KM)** |
| --- | --- | --- | --- | --- | --- |
| **KEYNOTE-522** | Clinical study report* | Clinical study report* | Clinical study report* | Clinical study report* | Clinical study report* |
| **Alliance (CALGB 40603)** | Sikov et al. 2015  Table 2 | https://clinicaltrials.gov/ct2/show/results/NCT00861705** | -- | https://clinicaltrials.gov/ct2/show/results/NCT00861705 | -- |
| **GeparSepto** | Untch et al. 2019 | Untch et al. 2019  Table 1 | Untch et al. 2019  Appendix Figure A2B | Untch et al. 2019  Table 1 | -- |
| **neoSTOP** | Sharma et al. 2021  Figure 1 | **Sharma et al. 2021**  **Figure 2A** | Sharma et al. 2021  Figure 2A | **Sharma et al. 2021**  **Figure 2B** | Sharma et al. 2021  Figure 2B |
| **BrighTNess** | Loibl et al. 2018  Figure 2A | Loibl et al. 2021  Slide 7 | Loibl et al. 2021  Slide 7 | Loibl et al. 2021  Slide 9 | Loibl et al. 2021  Slide 9 |

**Abbreviations:** EFS = event-free survival; HR = hazard ratio; KM = Kaplan-Meier; OS = overall survival; pCR = pathologic complete response.

**Notes: Bold** denotes HRs calculated from KM curves. Dashes (--) indicate where data were not reported.

*Provided by Merck & Co., Inc. **Reported as recurrence-free survival.

Additional Figure 1. Results of network meta-analysis for event-free survival based on time-varying hazard ratios (constant hazards with p_1_=0.5, p_2_=0)


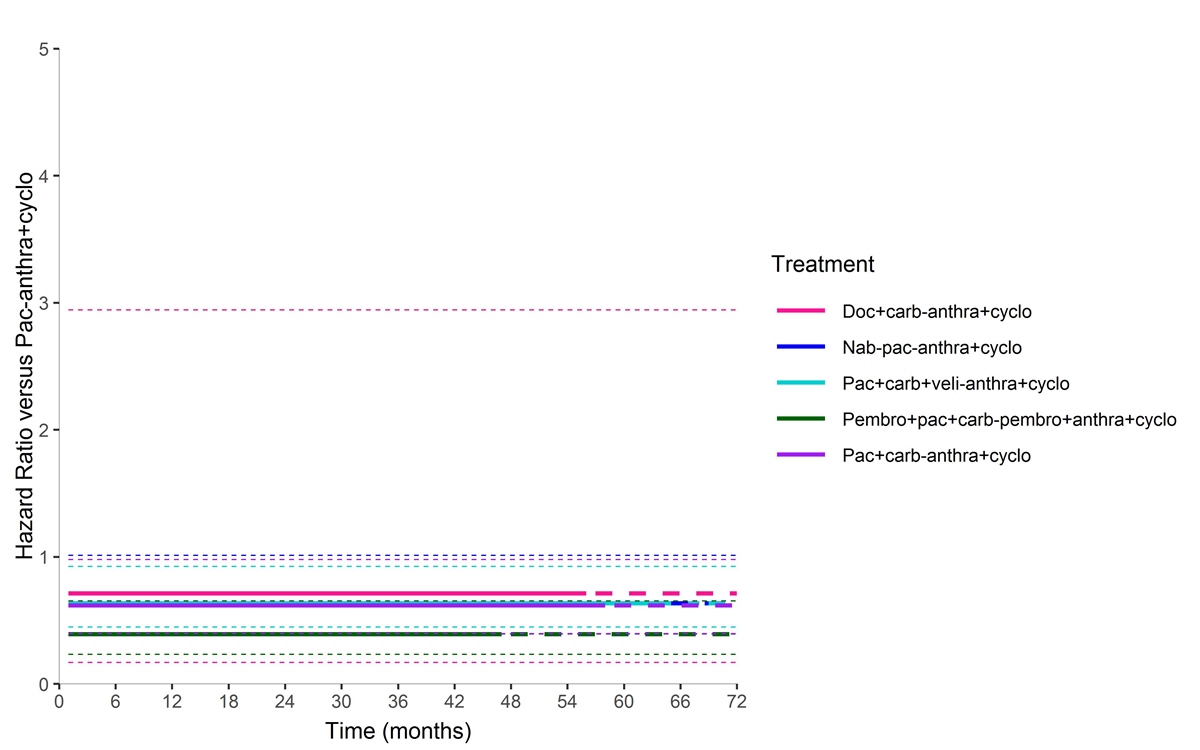


Additional Figure 2. Best-fitting model: Results of network meta-analysis for overall survival based on constant hazard ratio model with p_1_=0, p_2_=0


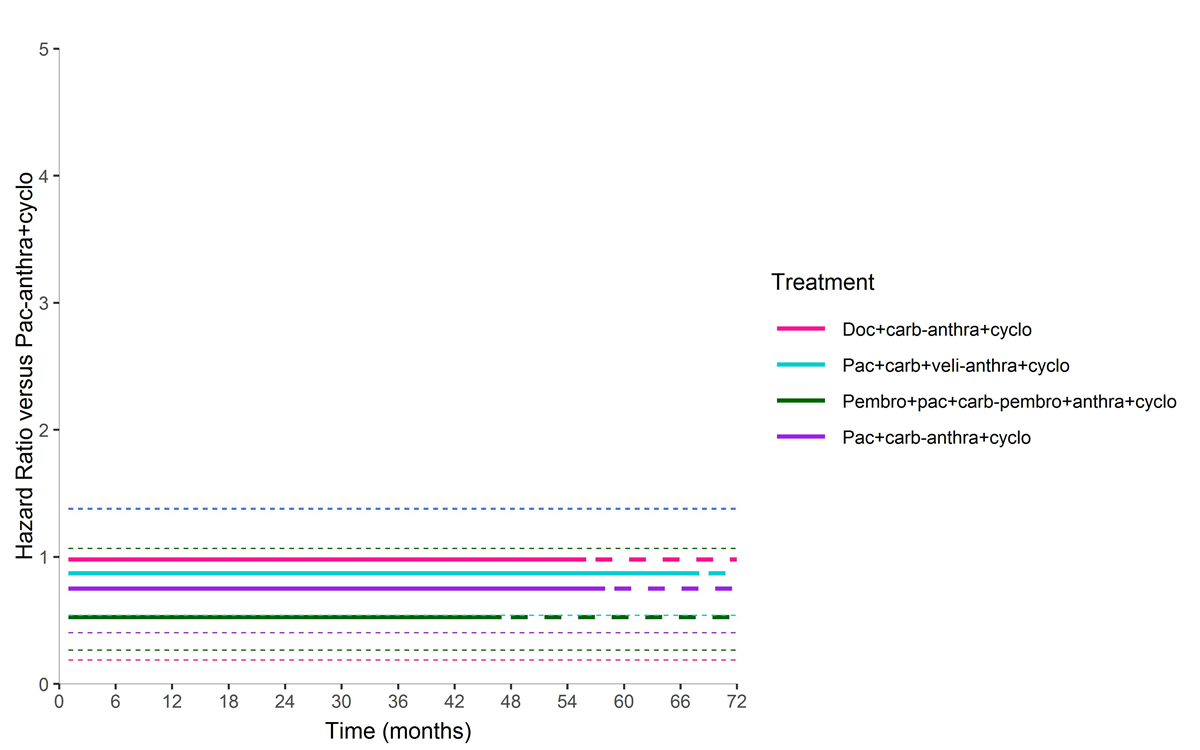


Additional Figure 3. Second best-fitting model: Results of network meta-analysis for overall survival based on second-order fractional polynomial model with p_1_=0, p_2_=0.5; scale and second shape


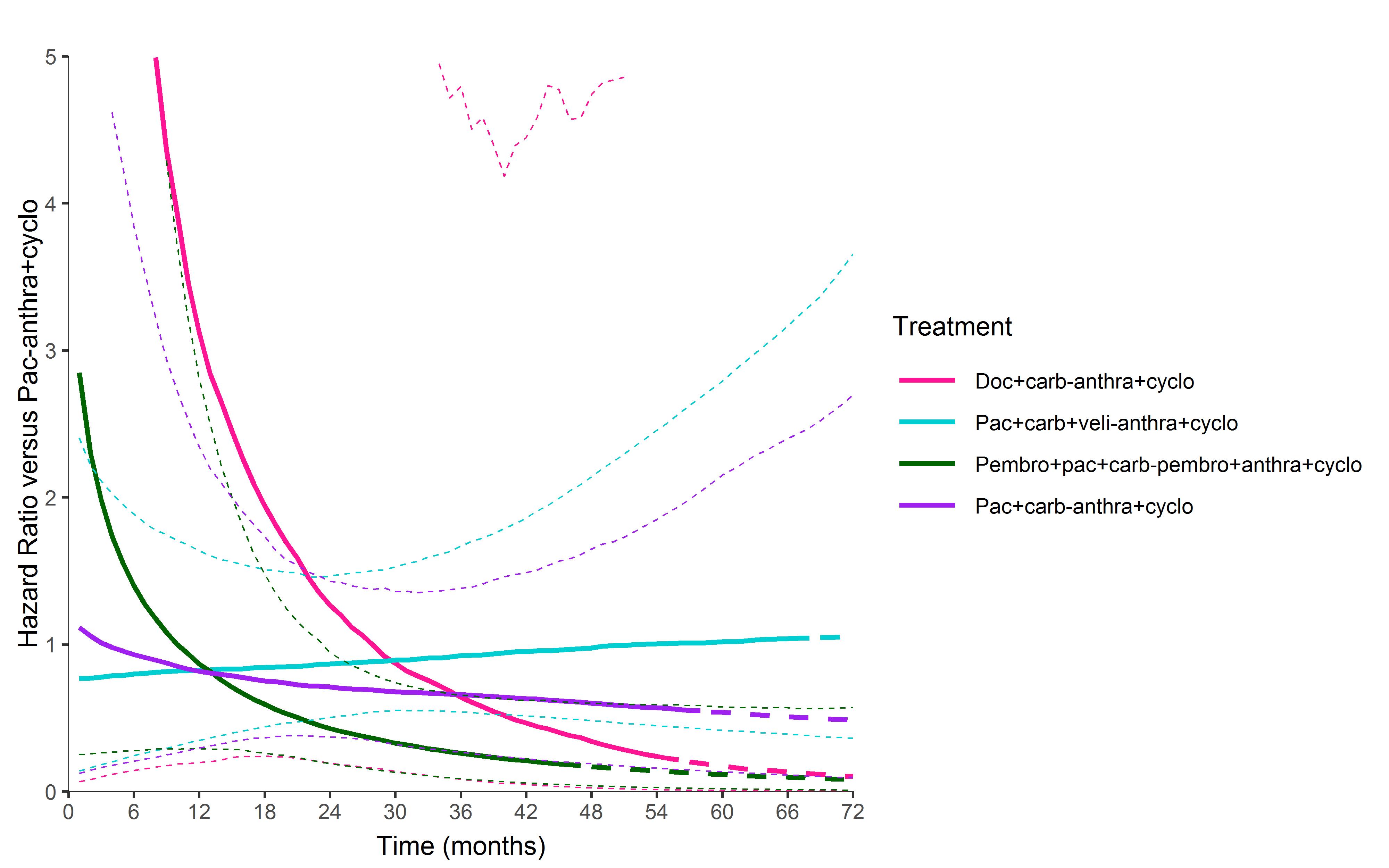


Additional Table 11. Model fit estimate for network meta-analysis for event-free survival with parametric survival models

| **Model** | **Dbar** | **pD** | **DIC** | **Zerods** |
| --- | --- | --- | --- | --- |
| **Constant HR with p_1_=0, p_2_=0.5** | **990.65** | **16.85** | **1007.50** | **Constant HR** |
| Second-order FP with p_1_=0, p_2_=0.5 | 989.32 | 21.39 | 1010.71 | Scale + first shape |
| Second-order FP with p_1_=0, p_2_=0.5 | 991.85 | 21.47 | 1013.32 | Scale + second shape |
| Second-order FP with p_1_=0, p_2_=0 | 992.90 | 22.34 | 1015.24 | Scale + first shape |
| Constant HR with p_1_=0, p_2_=0 | 995.07 | 20.75 | 1015.82 | Constant HR |
| Constant HR with p_1_=1, p_2_=0 | 999.96 | 17.16 | 1017.12 | Constant HR |
| Second-order FP with p_1_=0, p_2_=0 | 995.83 | 23.52 | 1019.35 | Scale + second shape |
| Constant HR with p_1_=0, p_2_=1 | 1001.23 | 18.33 | 1019.56 | Constant HR |
| Second-order FP with p_1_=0, p_2_=1 | 997.87 | 22.70 | 1020.57 | Scale + second shape |
| Second-order FP with p_1_=0, p_2_=1 | 1000.64 | 20.71 | 1021.35 | Scale + first shape |
| Second-order FP with p_1_=1, p_2_=0 | 1003.09 | 20.97 | 1024.06 | Scale + second shape |
| Constant HR with p_1_=1, p_2_=-0.5 | 1007.43 | 18.27 | 1025.70 | Constant HR |
| Second-order FP with p_1_=1, p_2_=-0.5 | 1005.64 | 21.39 | 1027.03 | Scale + first shape |
| Constant HR with p_1_=1, p_2_=-1 | 1014.33 | 14.71 | 1029.04 | Constant HR |
| Second-order FP with p_1_=0, p_2_=-1 | 1016.21 | 18.29 | 1034.50 | Scale + second shape |
| Second-order FP with p_1_=1, p_2_=-1 | 1012.49 | 22.66 | 1035.15 | Scale + second shape |
| Constant HR with p_1_=0, p_2_=-0.5 | 1020.83 | 14.47 | 1035.30 | Constant HR |
| Second-order FP with p_1_=0, p_2_=-0.5 | 1018.09 | 17.98 | 1036.07 | Scale + second shape |
| Second-order FP with p_1_=0, p_2_=-1 | 1021.70 | 14.62 | 1036.32 | Constant HR |
| Second-order FP with p_1_=1, p_2_=0 | 1009.09 | 29.53 | 1038.62 | Scale + first shape |
| Second-order FP with p_1_=0, p_2_=-0.5 | 1022.06 | 19.87 | 1041.93 | Scale + first shape |
| Constant HR with p_1_=1, p_2_=0.5 | 1023.89 | 18.21 | 1042.10 | Constant HR |
| Second-order FP with p_1_=0, p_2_=-1 | 1021.70 | 20.42 | 1042.12 | Scale + first shape |
| Second-order FP with p_1_=1, p_2_=0.5 | 1020.64 | 22.90 | 1043.54 | Scale + second shape |
| Second-order FP with p_1_=, p_2_=-1 | 1025.98 | 20.39 | 1046.37 | Scale + first shape |
| Second-order FP with p_1_=1, p_2_=-0.5 | 1021.06 | 29.53 | 1050.59 | Scale + second shape |
| Second-order FP with p_1_=1, p_2_=0.5 | 1040.30 | 31.65 | 1071.95 | Scale + first shape |
| Gompertz (first-order FP with p=1) | 1082.96 | 13.59 | 1096.55 | Constant HR |
| Gompertz (first-order FP with p=1) | 1081.28 | 16.63 | 1097.91 | Scale + first shape |
| Weibull (first-order FP with p=0) | 1107.90 | 13.16 | 1121.06 | Constant HR |
| Weibull (first-order FP with p=0) | 1108.49 | 17.48 | 1125.97 | Scale + first shape |
| Second-order FP with p_1_=1, p_2_=1 | 1443.91 | 23.57 | 1467.48 | Scale + first shape |
| Second-order FP with p_1_=1, p_2_=1 | 1536.76 | 36.24 | 15730 | Scale + second shape |
| Constant HR with p_1_=1, p_2_=1 | 1532.74 | 62.88 | 1595.62 | Constant HR |

**Abbreviations**: Dbar = posterior mean of the deviance; DIC = deviance information criterion; pD = effective number of parameters; FP = fractional polynomial; HR = hazard ratio; Zerods = scale parameter, first shape parameter, and second shape parameter.

**Notes:** **Bolded rows** indicate the best model fit.

**Additional Table 12. Estimated hazard ratios for event-free survival versus paclitaxel followed by anthracycline + cyclophosphamide at select time points based on time-varying hazard ratio assumption (constant hazard ratio with p_1_=0.5, p_2_=0)**

| **vs. Pac 🡪 anthra + cyclo** | **Time-varying HR (95% CrI)** | | | | | | | | | | | | | |
| --- | --- | --- | --- | --- | --- | --- | --- | --- | --- | --- | --- | --- | --- | --- |
|  | **3 months** | **6 months** | **9 months** | **12 months** | **18 months** | **24 months** | **30 months** | **36 months** | **42 months** | **48 months** | **54 months** | **60 months** | **66 months** | **72 months** |
| **Doc + carb 🡪 anthra + cyclo** | 0.71  (0.17, 2.94) | 0.71  (0.17, 2.94) | 0.71  (0.17, 2.94) | 0.71  (0.17, 2.94) | 0.71  (0.17, 2.94) | 0.71  (0.17, 2.94) | 0.71  (0.17, 2.94) | 0.71  (0.17, 2.94) | 0.71  (0.17, 2.94) | 0.71  (0.17, 2.94) | 0.71  (0.17, 2.94) | 0.71  (0.17, 2.94) | 0.71  (0.17, 2.94) | 0.71  (0.17, 2.94) |
| **Nab-pac 🡪 anthra + cyclo** | 0.63  (0.39, 1.01) | 0.63  (0.39, 1.01) | 0.63  (0.39, 1.01) | 0.63  (0.39, 1.01) | 0.63  (0.39, 1.01) | 0.63  (0.39, 1.01) | 0.63  (0.39, 1.01) | 0.63  (0.39, 1.01) | 0.63  (0.39, 1.01) | 0.63  (0.39, 1.01) | 0.63  (0.39, 1.01) | 0.63  (0.39, 1.01) | 0.63  (0.39, 1.01) | 0.63  (0.39, 1.01) |
| **Pac + carb + veli 🡪 anthra + cyclo** | **0.63  (0.45, 0.92)** | **0.63  (0.45, 0.92)** | **0.63  (0.45, 0.92)** | **0.63  (0.45, 0.92)** | **0.63  (0.45, 0.92)** | **0.63  (0.45, 0.92)** | **0.63  (0.45, 0.92)** | **0.63  (0.45, 0.92)** | **0.63  (0.45, 0.92)** | **0.63  (0.45, 0.92)** | **0.63  (0.45, 0.92)** | **0.63  (0.45, 0.92)** | **0.63  (0.45, 0.92)** | **0.63  (0.45, 0.92)** |
| **Pembro + pac + carb 🡪 pembro + anthra + cyclo** | **0.39  (0.23, 0.65)** | **0.39  (0.23, 0.65)** | **0.39  (0.23, 0.65)** | **0.39  (0.23, 0.65)** | **0.39  (0.23, 0.65)** | **0.39  (0.23, 0.65)** | **0.39  (0.23, 0.65)** | **0.39  (0.23, 0.65)** | **0.39  (0.23, 0.65)** | **0.39  (0.23, 0.65)** | **0.39  (0.23, 0.65)** | **0.39  (0.23, 0.65)** | **0.39  (0.23, 0.65)** | **0.39  (0.23, 0.65)** |
| **Pac + carb 🡪 anthra + cyclo** | **0.62  (0.39, 0.98)** | **0.62  (0.39, 0.98)** | **0.62  (0.39, 0.98)** | **0.62  (0.39, 0.98)** | **0.62  (0.39, 0.98)** | **0.62  (0.39, 0.98)** | **0.62  (0.39, 0.98)** | **0.62  (0.39, 0.98)** | **0.62  (0.39, 0.98)** | **0.62  (0.39, 0.98)** | **0.62  (0.39, 0.98)** | **0.62  (0.39, 0.98)** | **0.62  (0.39, 0.98)** | **0.62  (0.39, 0.98)** |

**Abbreviations:** Anthra = anthracycline; Carb = carboplatin; Crl = credible interval; Cyclo = cyclophosphamide; Doc = docetaxel; EFS = event-free survival; HR = hazard ratio;

Nab-pac = nab-paclitaxel; Pac = paclitaxel; Pembro = pembrolizumab; Veli = veliparib.

**Notes:** Anthracycline includes doxorubicin and epirubicin, which were assumed to be equivalent. Arrows (**-->**) indicate where treatment was administered sequentially. Treatments to the left of the arrow were administered first. Cells shaded in grey indicate estimates based on model extrapolations; model presented is p_1_=0, p_2_=0.5, constant HR, fixed-effects; All **bolded** values are statistically significant at the 0.05 significance level.

Additional Table 13. Basic parameter estimates of constant hazard ratio model with p_1_=0.5, p_2_=0 for event-free survival

| **Treatment** | **d0_estimate** | **d0_var** |
| --- | --- | --- |
| Pac🡪anthra+cyclo | Reference | Reference |
| Doc+carb🡪anthra+cyclo | -0.33976 | 0.54033 |
| Nab-pac🡪anthra+cyclo | -0.45604 | 0.05731 |
| Pac+carb+veli🡪anthra+cyclo | -0.45680 | 0.03408 |
| Pembro+pac+carb🡪pembro+anthra+cyclo🡪adjuvant pembro | -0.94226 | 0.06673 |
| Pac+carb🡪anthra+cyclo | -0.48248 | 0.05208 |

**Abbreviations:** Anthra = anthracycline; Carb = carboplatin; Cyclo = cyclophosphamide; Doc = docetaxel; EFS = event-free survival; KM = Kaplan-Meier; Nab-pac = nab-paclitaxel; Pac = paclitaxel; Pembro = pembrolizumab; Veli = veliparib.

**Notes:** Anthracycline includes doxorubicin and epirubicin, which were assumed to be equivalent. Arrows (**🡪**) indicate where treatment was administered sequentially. Treatments to the left of the arrow were administered first.

Additional Table 14. Model fit estimate for network meta-analysis for overall survival with parametric survival models

| **Model** | **Dbar** | **pD** | **DIC** | **Zerods** |
| --- | --- | --- | --- | --- |
| **Constant HR with p_1_=0, p_2_=0** | **612.27** | **11.10** | **623.37** | **Constant HR** |
| **Second-order FP with p_1_=0, p_2_=0.5** | **609.42** | **16.38** | **625.80** | **Scale + second shape** |
| Constant HR with p**_1_=**0, p_2_=0.5 | 613.53 | 12.75 | 626.28 | Constant HR |
| Second-order FP with p**_1_=**0, p_2_=0 | 612.70 | 17.21 | 629.91 | Scale + second shape |
| Second-order FP with p**_1_=**0, p_2_=0.5 | 611.75 | 18.81 | 630.56 | Scale + first shape |
| Second-order FP with p**_1_=**1, p_2_=-0.5 | 615.66 | 15.68 | 631.34 | Scale + first shape |
| Constant HR with p**_1_=**0, p_2_=1 | 618.43 | 13.03 | 631.46 | Constant HR |
| Constant HR with p**_1_=**1, p_2_=-0.5 | 621.39 | 11.29 | 632.68 | Constant HR |
| Second-order FP with p**_1_=**0, p_2_=0 | 615.88 | 19.63 | 635.51 | Scale + first shape |
| Second-order FP with p**_1_=**1, p_2_=0 | 620.82 | 16.54 | 637.36 | Scale + first shape |
| Second-order FP with p**_1_=**1, p_2_=-0.5 | 626.88 | 12.55 | 639.43 | Scale + second shape |
| Second-order FP with p**_1_=**0, p_2_=-0.5 | 628.70 | 12.86 | 641.56 | Scale + second shape |
| Constant HR with p**_1_=**0, p_2_=-0.5 | 630.35 | 11.23 | 641.58 | Constant HR |
| Constant HR p**_1_=**0, p_2_=-1 | 630.23 | 11.48 | 641.71 | Constant HR |
| Second-order FP with p**_1_=**0, p_2_=-1 | 629.07 | 12.77 | 641.84 | Scale + second shape |
| Second-order FP with p**_1_=**0, p_2_=-1 | 627.34 | 15.59 | 642.93 | Scale + first shape |
| Second-order FP with p**_1_=**0, p_2_=-0.5 | 628.06 | 15.13 | 643.19 | Scale + first shape |
| Second-order FP with p**_1_=**0, p_2_=1 | 627.27 | 16.25 | 643.52 | Scale + second shape |
| Second-order FP with p**_1_=**0, p_2_=1 | 628.94 | 17.27 | 646.21 | Scale + first shape |
| Second-order FP with p**_1_=**1, p_2_=0 | 626.29 | 20.01 | 646.30 | Scale + second shape |
| Second-order FP with p**_1_=**1, p_2_=-1 | 632.32 | 14.56 | 646.88 | Scale + second shape |
| Constant HR p**_1_=**1, p_2_=0 | 633.03 | 15.50 | 648.53 | Constant HR |
| Second-order FP with p**_1_=**1, p_2_=-1 | 634.60 | 16.21 | 650.81 | Scale + first shape |
| Second-order FP with p**_1_=**1, p_2_=0.5 | 648.04 | 13.60 | 661.64 | Scale + second shape |
| Constant HR with p**_1_=**1, p_2_=-1 | 641.78 | 22.39 | 664.17 | Constant HR |
| Weibull (first-order FP with p=0) | 661.68 | 9.87 | 671.55 | Constant HR |
| Weibull (first-order FP with p=0) | 659.75 | 13.02 | 672.77 | Scale + first shape |
| Gompertz (first-order FP with p=1) | 660.25 | 12.98 | 673.23 | Scale + first shape |
| Second-order FP with p**_1_=**1, p_2_=0.5 | 660.80 | 14.50 | 675.30 | Constant HR |
| Gompertz (first-order FP with p=1) | 665.48 | 11.56 | 677.04 | Constant HR |
| Second-order FP with p**_1_=**1, p_2_=0.5 | 659.92 | 20.21 | 680.13 | Scale + first shape |
| Second-order FP with p**_1_=**1, p_2_=1 | 897.10 | 12.06 | 909.16 | Scale + second shape |
| Second-order FP with p**_1_=**1, p_2_=1 | 976.72 | 22.79 | 999.51 | Scale + first shape |
| Second-order FP with p**_1_=**1, p_2_=1 | 1163.36 | 47.65 | 1211.01 | Constant HR |

**Abbreviations**: Dbar = posterior mean of the deviance; DIC = deviance information criterion; pD = effective number of parameters; FP = fractional polynomial; HR = hazard ratio; Zerods = scale parameter, first shape parameter, and second shape parameter.

**Notes:** **Bolded rows** indicate the best model fits.

Additional Table 15. Best-fitting model for overall survival: Estimated hazard ratios versus paclitaxel followed by anthracycline + cyclophosphamide at select time points based on time-varying hazard ratio assumption (constant hazard ratio with p_1_=0, p_2_=0)

| **vs. Pac 🡪 anthra + cyclo** | **Time-varying HR (95% CrI)** | | | | | | | | | | | | | |
| --- | --- | --- | --- | --- | --- | --- | --- | --- | --- | --- | --- | --- | --- | --- |
|  | **3 months** | **6 months** | **9 months** | **12 months** | **18 months** | **24 months** | **30 months** | **36 months** | **42 months** | **48 months** | **54 months** | **60 months** | **66 months** | **72 months** |
| **Doc + carb 🡪 anthra + cyclo** | 0.98  (0.19, 6.45) | 0.98  (0.19, 6.45) | 0.98  (0.19, 6.45) | 0.98  (0.19, 6.45) | 0.98  (0.19, 6.45) | 0.98  (0.19, 6.45) | 0.98  (0.19, 6.45) | 0.98  (0.19, 6.45) | 0.98  (0.19, 6.45) | 0.98  (0.19, 6.45) | 0.98  (0.19, 6.45) | 0.98  (0.19, 6.45) | 0.98  (0.19, 6.45) | 0.98  (0.19, 6.45) |
| **Pac + carb + veli 🡪 anthra + cyclo** | 0.87  (0.54, 1.38) | 0.87  (0.54, 1.38) | 0.87  (0.54, 1.38) | 0.87  (0.54, 1.38) | 0.87  (0.54, 1.38) | 0.87  (0.54, 1.38) | 0.87  (0.54, 1.38) | 0.87  (0.54, 1.38) | 0.87  (0.54, 1.38) | 0.87  (0.54, 1.38) | 0.87  (0.54, 1.38) | 0.87  (0.54, 1.38) | 0.87  (0.54, 1.38) | 0.87  (0.54, 1.38) |
| **Pembro + pac + carb 🡪 pembro + anthra + cyclo 🡪 adjuvant pembro** | 0.52  (0.26, 1.06) | 0.52  (0.26, 1.06) | 0.52  (0.26, 1.06) | 0.52  (0.26, 1.06) | 0.52  (0.26, 1.06) | 0.52  (0.26, 1.06) | 0.52  (0.26, 1.06) | 0.52  (0.26, 1.06) | 0.52  (0.26, 1.06) | 0.52  (0.26, 1.06) | 0.52  (0.26, 1.06) | 0.52  (0.26, 1.06) | 0.52  (0.26, 1.06) | 0.52  (0.26, 1.06) |
| **Pac + carb 🡪 anthra + cyclo** | 0.75  (0.40, 1.37) | 0.75  (0.40, 1.37) | 0.75  (0.40, 1.37) | 0.75  (0.40, 1.37) | 0.75  (0.40, 1.37) | 0.75  (0.40, 1.37) | 0.75  (0.40, 1.37) | 0.75  (0.40, 1.37) | 0.75  (0.40, 1.37) | 0.75  (0.40, 1.37) | 0.75  (0.40, 1.37) | 0.75  (0.40, 1.37) | 0.75  (0.40, 1.37) | 0.75  (0.40, 1.37) |

**Abbreviations:** Anthra = anthracycline; Carb = carboplatin; Crl = credible interval; Cyclo = cyclophosphamide; Doc = docetaxel; HR = hazard ratio; Nab-pac = nab-paclitaxel; OS = overall survival; Pac = paclitaxel; Pembro = pembrolizumab; Veli = veliparib.

**Notes:** Anthracycline includes doxorubicin and epirubicin, which were assumed to be equivalent. Arrows (**-->**) indicate where treatment was administered sequentially. Treatments to the left of the arrow were administered first. Cells shaded in grey indicate estimates based on model extrapolations; model presented is p_1_=0, p_2_=0, constant HR, fixed-effects; All **bolded** values are statistically significant at the 0.05 significance level.

**Additional Table 16. Best-fitting model for overall survival: Basic parameter estimates of constant hazard ratio model with p_1_=0, p_2_=0**

| **Treatment** | **d0_estimate** | **d0_var** |
| --- | --- | --- |
| Pac🡪anthra+cyclo | Reference | Reference |
| Doc+carb🡪anthra+cyclo | -0.02304 | 0.80207 |
| Pac+carb+veli🡪anthra+cyclo | -0.13949 | 0.05712 |
| Pembro+pac carb🡪pembro+anthra+cyclo🡪adjuvant pembro | -0.64493 | 0.12532 |
| Pac+carb🡪anthra+cyclo | -0.28944 | 0.09410 |

**Abbreviations:** Anthra = anthracycline; Carb = carboplatin; Cyclo = cyclophosphamide; Doc = docetaxel; Nab-pac = nab-paclitaxel; OS = overall survival; Pac = paclitaxel; Pembro = pembrolizumab; Veli = veliparib.

**Notes:** Anthracycline includes doxorubicin and epirubicin, which were assumed to be equivalent. Arrows (**-->**) indicate where treatment was administered sequentially. Treatments to the left of the arrow were administered first.

Additional Table 17. Second best-fitting model for overall survival: Estimated hazard ratios versus paclitaxel followed by anthracycline + cyclophosphamide at select time points based on time-varying hazard ratio assumption (second-order fractional polynomial with p_1_=0, p_2_=0.5)

| **vs. Pac 🡪 anthra + cyclo** | **Time-varying HR (95% CrI)** | | | | | | | | | | | | | |
| --- | --- | --- | --- | --- | --- | --- | --- | --- | --- | --- | --- | --- | --- | --- |
|  | **3 months** | **6 months** | **9 months** | **12 months** | **18 months** | **24 months** | **30 months** | **36 months** | **42 months** | **48 months** | **54 months** | **60 months** | **66 months** | **72 months** |
| **Doc + carb 🡪 anthra + cyclo** | 11.20  (0.10, 2158.16) | 6.52  (0.14, 639.89) | 4.36  (0.18, 206.53) | 3.12  (0.20, 88.83) | 1.94  (0.24, 29.74) | 1.27  (0.19, 12.20) | 0.87  (0.14, 6.20) | 0.64  (0.08, 4.80) | 0.47  (0.05, 4.45) | 0.34  (0.02, 4.74) | 0.24  (0.01, 5.40) | 0.17  (0.01, 6.77) | 0.13  (0.00, 8.82) | 0.10  (0.00, 11.10) |
| **Pac + carb + veli 🡪 anthra + cyclo** | 0.78  (0.18, 2.11) | 0.80  (0.24, 1.89) | 0.81  (0.29, 1.75) | 0.82  (0.35, 1.64) | 0.84  (0.44, 1.51) | 0.87  (0.50, 1.46) | 0.89  (0.55, 1.53) | 0.92  (0.54, 1.67) | 0.95  (0.51, 1.86) | 0.98  (0.48, 2.14) | 1.00  (0.45, 2.46) | 1.02  (0.42, 2.79) | 1.04  (0.39, 3.16) | 1.05  (0.36, 3.66) |
| **Pembro + pac + carb 🡪 pembro + anthra + cyclo 🡪 adjuvant pembro** | 1.98  (0.26, 14.88) | 1.40  (0.28, 7.28) | 1.08  (0.29, 4.29) | 0.87  (0.29, 2.81) | 0.59  (0.26, 1.48) | **0.43  (0.19, 0.94)** | **0.33  (0.13, 0.74)** | **0.26  (0.09, 0.65)** | **0.21  (0.06, 0.62)** | **0.17  (0.04, 0.60)** | **0.14  (0.03, 0.59)** | **0.11  (0.02, 0.57)** | **0.10  (0.01, 0.57)** | **0.08  (0.01, 0.57)** |
| **Pac + carb 🡪 anthra + cyclo** | 1.01  (0.16, 5.21) | 0.93  (0.21, 3.85) | 0.87  (0.25, 2.94) | 0.82  (0.29, 2.35) | 0.75  (0.36, 1.73) | 0.71  (0.37, 1.43) | 0.68  (0.32, 1.36) | 0.66  (0.27, 1.38) | 0.63  (0.22, 1.49) | 0.60  (0.19, 1.65) | 0.57  (0.16, 1.85) | 0.54  (0.13, 2.15) | 0.51  (0.11, 2.40) | 0.48  (0.09, 2.69) |

**Abbreviations:** Anthra = anthracycline; Carb = carboplatin; Crl = credible interval; Cyclo = cyclophosphamide; Doc = docetaxel; HR = hazard ratio;

Nab-pac = nab-paclitaxel; OS = overall survival; Pac = paclitaxel; Pembro = pembrolizumab; Veli = veliparib.

**Notes:** Anthracycline includes doxorubicin and epirubicin, which were assumed to be equivalent. Arrows (**-->**) indicate where treatment was administered sequentially. Treatments to the left of the arrow were administered first. Cells shaded in grey indicate estimates based on model extrapolations; model presented is p_1_=0, p_2_=0.5, scale and second shape, fixed-effects; All **bolded** values are statistically significant at the 0.05 significance level.

**Additional Table 18. Second best-fitting model for overall survival: Basic parameter estimates of second-order polynomial model with p_1_=0, p_2_=0.5**

| **Treatment** | **d0 estimate** | **d0 var** | **d1 estimate** | **d1 var** | **d01 corr** |
| --- | --- | --- | --- | --- | --- |
| **Pac🡪anthra+cyclo** | Reference | Reference | Reference | Reference | Reference |
| **Doc+carb🡪anthra+cyclo** | 3.65137 | 12.79390 | -0.70967 | 0.40811 | -0.96438 |
| **Pac+carb+veli🡪anthra+cyclo** | -0.30006 | 0.91478 | 0.03740 | 0.02951 | -0.96017 |
| **Pembro+pac+carb🡪 pembro+anthra+cyclo🡪adjuvant pembro** | 1.55438 | 2.22151 | -0.47950 | 0.08610 | -0.96312 |
| **Pac+carb🡪anthra+cyclo** | 0.24178 | 1.63561 | -0.11975 | 0.05902 | -0.96216 |

**Abbreviations:** Anthra = anthracycline; Carb = carboplatin; Cyclo = cyclophosphamide; Doc = docetaxel; Nab-pac = nab-paclitaxel; OS = overall survival; Pac = paclitaxel; Pembro = pembrolizumab; Veli = veliparib.

**Notes:** Anthracycline includes doxorubicin and epirubicin, which were assumed to be equivalent. Arrows (**-->**) indicate where treatment was administered sequentially. Treatments to the left of the arrow were administered first.

Additional Figure 4. Network of evidence for event-free survival and overall survival including CALGB 40603 (Alliance). Arrows (🡪) indicate where treatment was administered sequentially, with treatments to the left of the arrow administered first. The orange circle denotes the primary treatment regimen of interest. Anthra includes doxorubicin and epirubicin, which were assumed to be equivalent. Anthra = anthracycline; bev = bevacizumab; carb = carboplatin; cyclo = cyclophosphamide; doc = docetaxel; nab-pac = nab-paclitaxel; pac = paclitaxel; pembro = pembrolizumab; veli = veliparib.


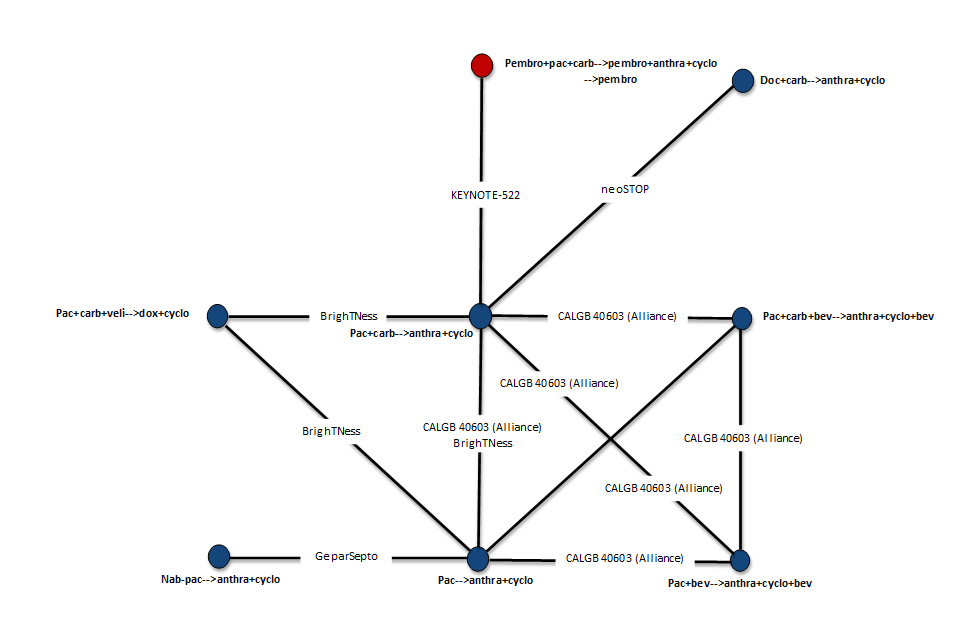


Additional Table 19. Results of network-analysis for event-free survival including CALGB 40603 (Alliance)

| **Intervention** | **Pac🡪anthra+cyclo** | **Pac+carb🡪**  **anthra+cyclo** | **Doc+carb🡪**  **anthra+cyclo**) | **Nab-pac🡪**  **anthra+cyclo** | **Pac+bev🡪**  **anthra+cyclo+bev** | **Pac+carb+bev🡪**  **anthra+cyclo+bev** | **Pac+carb+veli🡪**  **anthra+cyclo** | **Pembro+pac+carb🡪**  **pembro+anthra+cyclo🡪pembro** |
| --- | --- | --- | --- | --- | --- | --- | --- | --- |
| **Pac🡪anthra+cyclo** | **1** |  |  |  |  |  |  |  |
| **Pac+carb🡪**  **anthra+cyclo** | **0.68  (0.49, 0.96)** | **1** |  |  |  |  |  |  |
| **Doc+carb🡪**  **anthra+cyclo** | 0.79  (0.20, 3.09) | 1.16  (0.31, 4.37) | **1** |  |  |  |  |  |
| **Nab-pac🡪**  **anthra+cyclo** | **0.62  (0.39, 0.99)** | 0.91  (0.52, 1.63) | 0.78  (0.19, 3.22) | **1** |  |  |  |  |
| **Pac+bev🡪**  **anthra+cyclo+bev** | 0.84  (0.53, 1.33) | 1.22  (0.78, 1.93) | 1.05  (0.26, 4.23) | 1.35  (0.70, 2.60) | **1** |  |  |  |
| **Pac+carb+bev🡪**  **anthra+cyclo+bev** | 1.10  (0.68, 1.79) | 1.60  (0.99, 2.60) | 1.38  (0.33, 5.59) | 1.77  (0.90, 3.45) | 1.31  (0.78, 2.20) | **1** |  |  |
| **Pac+carb+veli🡪**  **anthra+cyclo** | **0.68  (0.47, 0.97)** | 0.99  (0.67, 1.46) | 0.85  (0.21, 3.33) | 1.09  (0.61, 1.95) | 0.81  (0.47, 1.39) | 0.62  (0.35, 1.08) | **1** | 1.58  (0.99, 2.51) |
| **Pembro+pac+carb🡪**  **pembro+anthra+cyclo🡪pembro** | **0.43  (0.28, 0.66)** | **0.63  (0.48, 0.82)** | 0.54  (0.14, 2.05) | 0.69  (0.37, 1.30) | **0.51  (0.30, 0.87)** | **0.39  (0.23, 0.68)** | 0.63  (0.40, 1.01) | **1** |

**Abbreviations:** Anthra = anthracycline; Bev = bevacizumab; Carb = carboplatin; CrI = credible interval; Cyclo = cyclophosphamide; DIC = deviance information criterion; Doc = docetaxel; ITT = intention-to-treat; HR = hazard ratio; Nab-pac = nab-paclitaxel; Pac = paclitaxel; Pembro = pembrolizumab; Veli = veliparib.

**Notes:** Each cell represents the comparison (HR and 95% CrI) of the row treatment versus the column treatment.

All **bolded** values are statistically meaningful at the 0.05 significance level. Deviance information criterion: 14.59; Deviance: 7.57.

Additional Table 20. Results of network-analysis for event-free survival including CALGB 40603 (Alliance)

| **Intervention** | **Pac🡪anthra+cyclo** | **Pac+carb🡪**  **anthra+cyclo** | **Doc+carb🡪**  **anthra+cyclo**) | **Nab-pac🡪**  **anthra+cyclo** | **Pac+bev🡪**  **anthra+cyclo+bev** | **Pac+carb+bev🡪**  **anthra+cyclo+bev** | **Pac+carb+veli🡪**  **anthra+cyclo** | **Pembro+pac+carb🡪**  **pembro+anthra+cyclo🡪pembro** |
| --- | --- | --- | --- | --- | --- | --- | --- | --- |
| **Pac🡪anthra+cyclo** | **1** | 1.49  (0.98, 2.29) | 1.15  (0.23, 5.43) | 1.34  (0.73, 2.52) | 1.22  (0.70, 2.10) | 0.84  (0.51, 1.36) | 1.19  (0.73, 1.92) | **2.07  (1.20, 3.59)** |
| **Pac+carb🡪**  **anthra+cyclo** | 0.67  (0.44, 1.02) | **1** | 0.77  (0.17, 3.36) | 0.90  (0.43, 1.91) | 0.81  (0.47, 1.41) | **0.56  (0.34, 0.91)** | 0.80  (0.47, 1.33) | 1.39  (0.99, 1.97) |
| **Doc+carb🡪**  **anthra+cyclo** | 0.87  (0.18, 4.26) | 1.30  (0.30, 6.04) | **1** | 1.17  (0.22, 6.44) | 1.06  (0.22, 5.32) | 0.73  (0.15, 3.59) | 1.04  (0.21, 5.31) | 1.80  (0.39, 8.78) |
| **Nab-pac🡪**  **anthra+cyclo** | 0.74  (0.40, 1.38) | 1.11  (0.52, 2.34) | 0.85  (0.16, 4.51) | **1** | 0.90  (0.40, 2.05) | 0.62  (0.28, 1.35) | 0.88  (0.40, 1.92) | 1.53  (0.68, 3.47) |
| **Pac+bev🡪**  **anthra+cyclo+bev** | 0.82  (0.48, 1.42) | 1.23  (0.71, 2.11) | 0.94  (0.19, 4.57) | 1.11  (0.49, 2.48) | **1** | 0.69  (0.40, 1.18) | 0.98  (0.49, 1.92) | 1.70  (0.89, 3.24) |
| **Pac+carb+bev🡪**  **anthra+cyclo+bev** | 1.19  (0.74, 1.95) | **1.78  (1.10, 2.91)** | 1.38  (0.28, 6.53) | 1.60  (0.74, 3.51) | 1.45  (0.85, 2.50) | **1** | 1.42  (0.76, 2.66) | **2.47  (1.35, 4.50)** |
| **Pac+carb+veli🡪**  **anthra+cyclo** | 0.84  (0.52, 1.36) | 1.25  (0.75, 2.11) | 0.96  (0.19, 4.68) | 1.14  (0.52, 2.49) | 1.02  (0.52, 2.04) | 0.71  (0.38, 1.32) | **1** | 1.73  (0.93, 3.25) |
| **Pembro+pac+carb🡪**  **pembro+anthra+cyclo🡪pembro** | **0.48  (0.28, 0.84)** | 0.72  (0.51, 1.01) | 0.56  (0.11, 2.54) | 0.65  (0.29, 1.47) | 0.59  (0.31, 1.13) | **0.40  (0.22, 0.74)** | 0.58  (0.31, 1.07) | **1** |

**Abbreviations:** Anthra = anthracycline; Bev = bevacizumab; Carb = carboplatin; CrI = credible interval; Cyclo = cyclophosphamide; DIC = deviance information criterion; Doc = docetaxel; ITT = intention-to-treat; HR = hazard ratio; Nab-pac = nab-paclitaxel; Pac = paclitaxel; Pembro = pembrolizumab; Veli = veliparib.

**Notes:** Each cell represents the comparison (HR and 95% CrI) of the row treatment versus the column treatment.

All **bolded** values are statistically meaningful at the 0.05 significance level. Deviance information criterion: 12.88; Deviance: 5.88.

**Additional References**

1. Gianni L, Mansutti M, Anton A, et al. Comparing Neoadjuvant Nab-paclitaxel vs Paclitaxel Both Followed by Anthracycline Regimens in Women With ERBB2/HER2-Negative Breast Cancer-The Evaluating Treatment With Neoadjuvant Abraxane (ETNA) Trial: A Randomized Phase 3 Clinical Trial. *JAMA Oncol*. Mar 1 2018;4(3):302-308. doi:10.1001/jamaoncol.2017.4612

2. Untch M, Jackisch C, Schneeweiss A, et al. Nab-paclitaxel versus solvent-based paclitaxel in neoadjuvant chemotherapy for early breast cancer (GeparSepto-GBG 69): a randomised, phase 3 trial. *Lancet Oncol*. Mar 2016;17(3):345-356. doi:10.1016/s1470-2045(15)00542-2

3. Untch M, Jackisch C, Schneeweiss A, et al. NAB-Paclitaxel Improves Disease-Free Survival in Early Breast Cancer: GBG 69–GeparSepto. *Journal of Clinical Oncology*. 2019/09/01 2019;37(25):2226-2234. doi:10.1200/JCO.18.01842

4. Mittendorf EA, Zhang H, Barrios CH, et al. Neoadjuvant atezolizumab in combination with sequential nab-paclitaxel and anthracycline-based chemotherapy versus placebo and chemotherapy in patients with early-stage triple-negative breast cancer (IMpassion031): a randomised, double-blind, phase 3 trial. *The Lancet*. 10 Oct 2020;396(10257):1090-1100. doi:<http://dx.doi.org/10.1016/S0140-6736%2820%2931953-X>

5. Mittendorf E, Harbeck N, Zhang H, et al. Abstract PD12-11: Patient-reported outcomes from the Phase III IMpassion031 trial of neoadjuvant atezolizumab + chemotherapy in early triple-negative breast cancer. *Cancer Research*. 2021;81(4_Supplement):PD12-11-PD12-11. doi:10.1158/1538-7445.Sabcs20-pd12-11

6. Schmid P, Cortes J, Dent R, et al. Event-free Survival with Pembrolizumab in Early Triple-Negative Breast Cancer. *New England Journal of Medicine*. 2022;386(6):556-567. doi:10.1056/NEJMoa2112651

7. Schmid P, Cortes J, Pusztai L, et al. Pembrolizumab for Early Triple-Negative Breast Cancer. Clinical Trial, Phase III; Multicenter Study; Randomized Controlled Trial; Research Support, Non-U.S. Gov't. *New England Journal of Medicine*. 02 27 2020;382(9):810-821. doi:<https://dx.doi.org/10.1056/NEJMoa1910549>

8. Dent R, Cortes J, Pusztai L, et al. 1O KEYNOTE-522 Asian subgroup: phase III study of neoadjuvant pembrolizumab (pembro) vs placebo (pbo) + chemotherapy (chemo) followed by adjuvant pembro vs pbo for early triple-negative breast cancer (TNBC). Journal: Conference Abstract. *Annals of oncology*. 2020;31:S1241-S1242.

9. Chen X, Ye G, Zhang C, et al. Superior outcome after neoadjuvant chemotherapy with docetaxel, anthracycline, and cyclophosphamide versus docetaxel plus cyclophosphamide: results from the NATT trial in triple negative or HER2 positive breast cancer. *Breast Cancer Res Treat*. Dec 2013;142(3):549-58. doi:10.1007/s10549-013-2761-1

10. Chen X, Ye G, Zhang C, Li X, Shen K. Non-anthracycline-containing docetaxel and cyclophosphamide regimen is associated with sustained worse outcome compared with docetaxel, anthracycline and cyclophosphamide in neoadjuvant treatment of triple negative and HER2-positive breast cancer patients: updated follow-up data from NATT study. *Chin J Cancer Res*. Dec 2016;28(6):561-569. doi:10.21147/j.issn.1000-9604.2016.06.02

11. Ademuyiwa FO, Gao F, Chen I, et al. Nci 10013 - A randomized phase 2study of neoadjuvant carboplatin and paclitaxel, with or without atezolizumab in triple negative breast cancer(TNBC). Journal: Conference Abstract. *Cancer research*. 2021;81(4 SUPPL)

12. Vriens BE, Aarts MJ, de Vries B, et al. Doxorubicin/cyclophosphamide with concurrent versus sequential docetaxel as neoadjuvant treatment in patients with breast cancer. *Eur J Cancer*. Oct 2013;49(15):3102-10. doi:10.1016/j.ejca.2013.06.012

13. ClinicalTrials.gov. Paclitaxel With or Without Carboplatin and/or Bevacizumab Followed by Doxorubicin and Cyclophosphamide in Treating Patients With Breast Cancer That Can Be Removed by Surgery. <https://clinicaltrials.gov/ct2/show/NCT00861705>

14. Sikov WM, Berry DA, Perou CM, et al. Impact of the addition of carboplatin and/or bevacizumab to neoadjuvant once-per-week paclitaxel followed by dose-dense doxorubicin and cyclophosphamide on pathologic complete response rates in stage II to III triple-negative breast cancer: CALGB 40603 (Alliance). *Journal of Clinical Oncology*. 01 Jan 2015;33(1):13-21. doi:<http://dx.doi.org/10.1200/JCO.2014.57.0572>

15. Sharma P, Kimler BF, O'Dea A, et al. Randomized Phase II Trial of Anthracycline-free and Anthracycline-containing Neoadjuvant Carboplatin Chemotherapy Regimens in Stage I–III Triple-negative Breast Cancer (NeoSTOP). *Clinical Cancer Research*. 2021;27(4):975-982. doi:10.1158/1078-0432.Ccr-20-3646

16. Loibl S, Sikov W, Huober J, et al. 119O Event-free survival (EFS), overall survival (OS), and safety of adding veliparib (V) plus carboplatin (Cb) or carboplatin alone to neoadjuvant chemotherapy in triple-negative breast cancer (TNBC) after≥ 4 years of follow-up: BrighTNess, a randomized phase III trial. *Annals of Oncology*. 2021;32:S408.

17. Loibl S, O'Shaughnessy J, Untch M, et al. Addition of the PARP inhibitor veliparib plus carboplatin or carboplatin alone to standard neoadjuvant chemotherapy in triple-negative breast cancer (BrighTNess): a randomised, phase 3 trial. *The Lancet Oncology*. 2018;19(4):497-509. doi:10.1016/S1470-2045(18)30111-6
